# Dairy Fats and Cardiovascular Disease: Do We Really Need to Be Concerned?

**DOI:** 10.3390/foods7030029

**Published:** 2018-03-01

**Authors:** Ronan Lordan, Alexandros Tsoupras, Bhaskar Mitra, Ioannis Zabetakis

**Affiliations:** 1 Department of Biological Sciences, University of Limerick, Limerick V94 T9PX, Ireland; ronan.lordan@ul.ie (R.L.); alexandros.tsoupras@ul.ie (A.T.); 2 Extrx Oy, Salmelantie 43, Sotkamo 88600, Finland; bhaskar.mitra@extrx.fi

**Keywords:** milk, cheese, yoghurt, *kefir*, cardiovascular diseases, inflammation, saturated fatty acids, atherosclerosis, cardiometabolic risk factors

## Abstract

Cardiovascular diseases (CVD) remain a major cause of death and morbidity globally and diet plays a crucial role in the disease prevention and pathology. The negative perception of dairy fats stems from the effort to reduce dietary saturated fatty acid (SFA) intake due to their association with increased cholesterol levels upon consumption and the increased risk of CVD development. Institutions that set dietary guidelines have approached dairy products with negative bias and used poor scientific data in the past. As a result, the consumption of dairy products was considered detrimental to our cardiovascular health. In western societies, dietary trends indicate that generally there is a reduction of full-fat dairy product consumption and increased low-fat dairy consumption. However, recent research and meta-analyses have demonstrated the benefits of full-fat dairy consumption, based on higher bioavailability of high-value nutrients and anti-inflammatory properties. In this review, the relationship between dairy consumption, cardiometabolic risk factors and the incidence of cardiovascular diseases are discussed. Functional dairy foods and the health implications of dairy alternatives are also considered. In general, evidence suggests that milk has a neutral effect on cardiovascular outcomes but fermented dairy products, such as yoghurt, *kefir* and cheese may have a positive or neutral effect. Particular focus is placed on the effects of the lipid content on cardiovascular health.

## 1. Introduction

Despite advances in improved primary prevention and medical treatment, cardiovascular diseases (CVD) are the leading cause of death and morbidity in Europe [1] and worldwide [2]. Every year CVD are responsible for 10,000 deaths in Ireland and 1.8 million in the European Union, due to coronary heart disease (CHD), stroke and related circulatory diseases [1,3]. Increasing evidence supports the pivotal function of nutrition in the development of chronic diseases, especially CVD [4]. Maladaptive diet and lifestyle are the dominant underlying cause of systemic inflammation, which is the core process that drives the development of atherosclerosis [4,5]. Diet and lifestyle are key modifiable risk factors for the prevention of CVD and thus have been the focus of intense research. Globally, striking differences in dietary habits and the rates of chronic disease exist. The identification and subsequent targeting of dietary factors with the greatest potential for reducing CVD, diabetes and obesity are of crucial scientific and public health importance [6]. 

Milk and dairy products are an important nutrient dense constituent of a healthy diet due to their capacity to provide essential vitamins, minerals, macronutrients and micronutrients important for growth, development and tissue maintenance. This is vital as globally 6 billion people consume milk and dairy products, the majority of them in developing countries [7]. Currently there are milks of varying fat content and milks with varying vitamin and mineral content (often fortified or enriched); there are also concentrated milks, high-protein milks, fermented milks and various other dairy products, including yoghurts and cheeses consumed worldwide. Dairy products are associated with many negative health effects due to previous observations relating to their saturated fatty acid (SFA) content, which may lead to increased low-density lipoprotein cholesterol (LDL) levels, thus an increased risk of cardiovascular disease [8]. There was also a strong correlation evident between dairy fat consumption and coronary heart disease in an early study [9]. However, recent findings have indicated that the link between SFA and CVD may be less clear than previously assumed. Foods are composed of an array of saturated and unsaturated fatty acids, each of which may differentially affect lipoprotein metabolism, as well as contribute significant quantities of other nutrients that may alter CVD risk [4,10]; these include phospholipids, milk proteins, calcium and vitamin D, which have been reviewed in recent literature [4,11]. In addition, recent research trends indicate that dairy products have a neutral [12,13,14] or even a positive effect on cardiovascular health [14,15,16,17,18,19] contrary to previous assumptions [4]. Dairy products have also been associated with positive health benefits against diabetes [20], obesity [21,22,23] and metabolic syndrome [23,24]. The way consumers obtain nutrition and dietary information has substantially changed. There is conflicting advice online with regards to every food type and many consumers are more confused now than ever [25]. Discrepancies in dietary advice due to the increased influence of the food industry, social media and ‘fad diets,’ which are often endorsed by celebrities and ‘diet gurus’ [26], among other modern phenomena has damaged consumer confidence in the nutritional value of dairy products. This review aims to assess the effect of dairy products on cardiometabolic health.

## 2. Dietary Guidelines and Dairy Product Consumption

Dairy intake is increasing worldwide, yet theoretically dairy products could both increase and decrease cardiometabolic risk factors. Dairy products have been associated with several cardiometabolic benefits, however the active constituents have not yet been established. Initially, dietary guidelines formulated in the 1980s, demonised dairy products due to their high SFA, cholesterol and calorie content [4]. The rise of the lipid hypothesis led most scientific organisations and dietary guidelines to recommend low-fat (1%) or non-fat dairy consumption, as a result of their characteristically high SFA content [27,28]. Now most countries recommend the consumption of dairy products and when amounts are specified, recommendations are typically for 2 or 3 servings per day [29]. Although often, as occurs in the UK, general recommendations are made with no specific amount mentioned [29]. There remains inconsistent health advice to consumers with many authorities recommending three to four servings a day [27] and labelling dairy as a ‘superfood’ [30]. On the other hand, some dietary guidelines recommend the complete avoidance of full-fat dairy products [31], others recommend that the consumption of dairy products be limited as much as possible [32]. However, as research advances, some countries such as Australia have revised their dietary guidelines and included dairy products but preferably consumption of low-fat as opposed to full-fat products is advised [33]. Notably, butter and cream do not fall into the category of dairy products in some dietary recommendations due to their significant contribution of fat to the diet [34].

Nevertheless, dairy products are nutrient dense, providing a wide range of crucial vitamins (A, B6, B12, D and K), minerals (calcium, iodine, magnesium, potassium, phosphorus and zinc), fats, proteins and other microconstituents [35,36], which are otherwise difficult to obtain in diets with limited use of dairy products [29]. In particular, dairy products can provide up to 60% of the recommended daily allowance (RDA) of calcium [37]. Furthermore, fermented dairy products are an excellent source of vitamin K, a fat-soluble vitamin [36] that will be discussed further in Section 4. 

In addition, fermented dairy products, such as yoghurt have a positive effect on intestinal microbiota. Therefore, dairy products generally form an integral role in the dietary guidelines of many countries. However, not all dairy products are created nutritionally equal. For instance, cheeses are often salted, which contributes to high sodium intake. Soft cheeses typically contain less calcium as the curd is formed with acid, some calcium is lost to the whey. The fat content of dairy products can vary greatly, because of the type of milk, degree of fat removal, the animals condition, diet and the milk processing [4,29]. Often ice-cream and similar dessert products are included as dairy products in dietary guidelines. The nutritional quality of these products is diluted by the addition of sugar and fats [29], in particular high amounts of vegetable fats like coconut oil and palm oil, that have questionable positive and negative effects on cardiovascular health due to their high lauric acid and palmitic acid content respectively [38,39,40]. Therefore, ice cream and dairy desserts that contain high levels of vegetable oils should be approached with caution and not considered in dairy research or as part of dairy products in dietary guidelines.

## 3. Saturated Fat, Cholesterol and Dairy Products

SFA and cholesterol have formed the basis of the ‘lipid hypothesis’ for CVD development. Consumption of full-fat dairy products was reduced and either substituted by a reduced fat version or intake was restricted based on government dietary guidelines. However, recent perspectives have determined more complex mechanisms for the underlying causes, initiation and development of CVD, that do not necessarily indicate that SFA or cholesterol levels are culpable. This section discusses the role of SFA and cholesterol on cardiovascular health, with respect to dairy products. 

### 3.1. Saturated Fat

Cholesterol levels are considered the most important risk factor for CVD development and so milk and dairy products theoretically have detrimental effects on cholesterol concentrations due to their SFA content [41]. For decades, it has been assumed that SFA (containing 12–18 carbon atoms) consumption undermines cardiovascular health [42,43,44,45]. By the 1960s, the ideologies of the lipid hypothesis were gathering momentum and the low-fat diet began to be touted for high-risk heart patients and for people’s health in general. Dietary advice to limit the consumption of SFA appeared as early as 1961 [46] and the rationale supporting these views was that SFA increased cholesterol levels, which in turn increases the risk of CVD development [47]. However, by the 1980s, the low-fat dietary approach became more popular and was promoted by the food industry, medical doctors, governments and popular health media [48]. Dairy products are high in SFA and their consumption has long been thought to contribute to CVD development [4]. As dairy products are the only food group composed of more saturated than unsaturated fat, it became apparent that they may be detrimental to cardiovascular health. Thus, it was proposed that low-fat or non-fat dairy products be advised to reduce the risk of developing CVD. However, the extent and precise nature of the role of saturated fat in CVD development and progression are being re-examined [43,49]. In fact, it has been proposed by many, that SFA should no longer be considered a single nutrient group but instead should be considered as individual molecules with specific functions of their own [4,50]. In animal fats, SFA are generally located in the *sn*-2 position of the glycerol backbone, while monounsaturated fatty acids and polyunsaturated fatty acids generally occupy the *sn*-1 and *sn*-3 positions, which induce specific effects on lipoprotein metabolism and atherogenesis [51]. When SFA is reduced in the diet, it is replaced by another macronutrient such as carbohydrate or protein. These diets do not necessarily demonstrate a decreased risk of CVD. Generally it is accepted that the replacement of saturated fat by polyunsaturated fatty acids (PUFA) in the diet is associated with a reduced risk of CVD [52], however this is still disputed by some due to insufficient evidence [53,54], which has been highlighted in recent reviews [53,55]. While the substitution of SFA with monounsaturated fatty acids or high-glycaemic index food remains less clear [52,56]. 

The proportion of SFA entering the food chain from milk and dairy products is controlled by industrial skimming, which reduces total milk fat. Other methods to reduce SFA include the alteration of the animal’s diet to include additional PUFA and monounsaturated fatty acids (MUFA), which replaces the SFA in the milk [4,57]. Initiatives include the supplementation of the bovine diet with an oleic acid-rich diet, through the addition of marine oils, plant oil and oilseeds as a novel means to reduce the intake of SFA [58]. In a recent study plant oils were supplemented to a grass silage-based bovine diet, which managed to reduce ruminal CH_4_ emission and milk saturated fatty acids but also increased the proportion of unsaturated fatty acids and total conjugated linoleic acid within the milk [59], thus possibly creating a feed that sustainably improves the lipid profile of the milk. Dairy products contain short, medium and long-chain SFA, which affect cardiovascular health differently. It is apparent that lauric (12:0), myristic (14:0) and palmitic (16:0) have adverse effects on LDL, which is considered an important risk factor for CVD [60]. When compared to carbohydrate consumption in humans, lauric acid, myristic acid and palmitic acid raised total cholesterol and LDL, whereas stearic acid (18:0) does not. SFA raises high-density lipoproteins (HDL) but these effects are greater as the fatty acid chain length decreases and so generally lauric acid has the most beneficial effect on cholesterol profile [61]. Based on these studies, lauric acid may be a heart-healthy SFA [56].

The effectiveness of the reduction of SFA in the diet to lower CVD development has been the subject of major controversy and debate that reignites regularly [56]. The weaknesses in the data [62,63], the complex disease pathology, the numerous risk factors and the inadequate reliance on single biomarkers to assess CVD risk [52,56] have highlighted a number of discrepancies in the ‘lipid hypothesis’. Several meta-analyses and systematic reviews further cast doubt over the effect of SFA on CVD outcomes [49,64], particularly the controversial PURE study, which assessed the dietary intake of 135,335 subjects from 18 countries across five continents for 7.4 year and found that total fat and types of fat were not associated with CVD, myocardial infarction, or CVD mortality. Further still, SFA was inversely associated with stroke [65]. The PURE study investigators state that advice to restrict SFA “is largely based on selective emphasis on some observational and clinical data, despite the existence of several randomised trials and observational studies that do not support these conclusions” [65]. 

### 3.2. Dietary Cholesterol

Dairy products contain approximately 80 mg/110 g dietary cholesterol. However, the previous notion that dietary cholesterol may increase the risk of developing CVD is currently under debate, since it is not well-supported in the literature. It is thought that the lack of evidence may depend on an individual predisposition to synthesise versus absorb cholesterol [66,67]. Therefore, dietary guidelines in the US [68], which recommended that cholesterol consumption be kept below 300 mg/day have come into question [66]. These recommendations were based on poor scientific evidence, apart from a known association between saturated fat and cholesterol and specific animal studies where cholesterol was fed in amounts far exceeding normal intakes [66]. In contrast, Canada [69], Ireland [28], the UK [70], Korea [71], New Zealand [72] and other Asian and European countries do not prescribe an upper limit for dietary cholesterol [66]. Epidemiological studies have shown that increasing dietary cholesterol levels are not correlated with increased risk of CVD [73,74,75]. However, clinical studies demonstrate that dietary cholesterol may increase serum LDL in certain individuals (hyper-responders); this is generally accompanied by increases in HDL. Therefore, the LDL/HDL ratio in hyper-responders remains the same [76,77]. The size of circulating LDL particles is also a major risk factor for CVD, which dietary cholesterol has the ability to reduce [66]. Controversy also surrounds the consumption of eggs due to their high cholesterol content, even though egg intake is associated with a number of benefits against CVD [53,78]. However it was researchers in this field that highlighted the need to review dietary cholesterol recommendations [66]. 

The effect of dairy consumption on cholesterolaemia has exhibited varied results. An early study demonstrated that high milk intake in an African Maasai ethnic group was inversely correlated with blood cholesterol levels [79]. Later, these observations were confirmed by others [80,81,82] and it was hypothesised that that this effect was a result of intestinal microbial fermentation of indigestible carbohydrates, that could alter cholesterol synthesis and disrupt enterohepatic circulation, thus lowering cholesterolaemia [83]. Studies eventually expanded to various dairy products; one examined the effects of an isoenergetic (20% of total calories, normalised for casein and lactose) provision of milk (2164 mL), butter (93 g) and cheese (305 g) administered in three sessions over three weeks. The authors found that cheese did not significantly raise LDL levels. In contrast, whole milk raised serum LDL levels similarly to butter [84]; results that were subsequently confirmed by Biong et al. who also demonstrated that consumption of cheese induced a lower increase of serum LDL cholesterol levels in 22 participants, in contrast to an identical mass of butter (42 g) consumed [85]. It was hypothesised that the different effects were a result of different calcium contents between the dairy products. Another study fed 40 g/day of either butter or mature cheddar cheese to 19 mildly hypercholesterolaemic participants four weeks in a randomised crossover trial [86]. They observed that total cholesterol and LDL cholesterol increased significantly in the butter group (*p* < 0.05) versus cheese, which has been observed previously [86]. The authors suggested that dietary advice surrounding cheese consumption should be revised. This was also questioned by Tholstrup et al. who suggested that modest amounts of cheese should be included in the diets of mildly hypercholesterolaemic participants [84]. Similar studies have observed similar results for cheese consumption. A recent study replaced 13% of the daily calorie intake with 47 g of butter or 143 g of cheese that possessed the same lipid content for six weeks in 49 participants. The results of the randomised crossover trial indicated that compared with the run-in period, cheese did not increase serum LDL cholesterol levels; rather as compared with butter, cheese induced a significantly lower increase in total (5.7%) and LDL (6.9%) cholesterol [87]. However, there was a lack of difference reported in the cholesterolaemic effects on diets containing full-fat milk and butter [41]. These observations have been observed in a recent study that compared the effect of equal amounts of SFA from butter and cheese intake in 92 overweight subjects [88]. Their results also indicated that consumption of SFA from butter and cheese had similar effects on HDL levels but differential effects on LDL levels, which they suggest may be explained by the food-matrix effect. 

Several attempts have been made to elucidate the mechanisms surrounding the differential effects of cheese and butter on cholesterolaemia. One theory is that calcium intake may increase faecal excretion of bile acids that would cause a regeneration of bile acids from hepatic cholesterol and thereby result in a lowering of plasma cholesterol concentrations. It is thought that the higher calcium concentration in cheese combines with fatty acids in the intestine and forms insoluble detergents. In addition, hydrophobic aggregates can form between phosphorus and bile acids that can be excreted and measured; these observations are indicative of reduced fat absorption [89,90]. Higher-fat faecal excretion has been observed in cheese groups versus butter groups [87]. However, a randomised controlled crossover dietary intervention study assessing bile acid and calcium concentrations in faecal samples from humans after intake of cheese and butter in 23 participants, again replacing 13% of their of their daily calorie intake was conducted to confirm these observations [91]. After 6 weeks of the intervention, cheese resulted in higher amounts of calcium excreted in faeces compared to butter. However, no difference was observed in faecal bile acid output despite lower serum total, LDL and HDL cholesterol concentrations observed with cheese intake. Although well designed, it is unfortunate that the mechanisms responsible for the lowering of cholesterol concentrations with cheese compared to butter intake remain unresolved. It is also thought that the protein and probiotic content of cheese may contribute to the observed neutral effect on serum cholesterol [91]. In addition, butter is not necessarily a good comparator for studies examining cheese intake [34]. Also, there are several confounding variables that prevent the formation of any solid conclusions in relation to cheese and CVD risk due to the differential effect of individual cheese varieties that differ in macronutrient content, degree of fermentation and food matrix [10].

Butter was previously associated with negative CVD outcomes. However, growing uncertainty and changing views on the role of butter in CVD has been highlighted by many, particularly Time Magazine [92]. Butter is composed of mainly milk fat but can contain some proteins, water and sometimes added salt. Butter fat in general consistently raises plasma cholesterol concentrations, especially in hypercholesterolaemic individuals and so may pose a risk to cardiovascular health [86,88,93,94]. Butter consumption has been consistently associated with negative cardiovascular risk outcomes due to the focus on cholesterol levels, however the long-term effects of butter consumption on other major endpoints, such as all-cause mortality and CVD, are also not well-established. In 2014, a systematic review and meta-analysis that examined the effect of butter consumption in 636,151 subjects from 15 country-specific cohorts on the risk of CVD, type II diabetes mellitus (T2DM), and total mortality was published [95]. Pimpin and colleagues discovered that butter was weakly associated with all-cause mortality in (*N* = 9 country-specific cohorts; per 14 g/day: relative risk (RR) = 1.01, 95% confidence interval (CI) = 1.00–1.03, *p* = 0.045); was not significantly associated with any CVD (*N* = 4; RR = 1.00, 95% CI = 0.98–1.02; *p* = 0.704), coronary heart disease (*N* = 3; RR = 0.99, 95% CI = 0.96–1.03; *p* = 0.537), or stroke (*N* = 3; RR = 1.01, 95% CI = 0.98–1.03; *p* = 0.737) and was inversely associated with incidence of diabetes (*N* = 11; RR = 0.96, 95% CI = 0.93–0.99; *p* = 0.021) [95]. The association of butter and CVD may not be as clear cut as previously thought. There is a lack of published studies to conclusively label butter as positive or negative on cardiovascular health. Thus, future studies must examine the effects of butter consumption on CVD risk, inflammatory markers and other risk factors to put the butter debate to rest. 

### 3.3. Low-Fat Dairy products

Since the 1970s, dietary guidelines were based on the findings of the ‘Seven Countries Study’, which indicated that high intakes of SFA and cholesterol were correlated with CVD. In light of these findings, 1977 saw the first edition of ‘The Dietary Goals for the United States’. These guidelines were formed in an attempt to reduce the incidence of diet-related diseases such as CVD [96]. The guidelines specified several alterations that were believed to improve health, including the alteration of fat consumption for Americans. While these guidelines were well-intended, they promoted the overhaul of the food industry and the average American’s perception of a nutritious diet, eventually contributing to overall decline in health, an increased national obesity and CVD rate, rather than the projected opposite result [97]. Diets from the 1970s onwards began to rely upon highly processed foods, increased food consumption away from home and a greater use of edible oils and sugar sweetened beverages, which coincided with reduced physical activity and an increased sedentary lifestyle [97]. The recommendations initiated the production of heavily used vegetable oils, radically altering the dietary *n*-6/*n*-3 ratio and increasing the requirement for hydrogenated oils. The food industry used excess sugar in product development in order to accommodate for the loss of flavour due to the reduction of fat in processed products. In addition, the advertising of ‘low-fat’ food labels further convinced people that ‘low-fat’ was synonymous with ‘healthy’, increasing the intake of overly processed foods and decreasing health status [97]. In fact, the United States government advised the public to increase their intake of carbohydrate (6–11 servings) and to consume all fats sparingly, as illustrated in the food guide pyramid of 1992 [98].

Low-fat dairy products became popular and easy to produce in response to consumer’s needs for reduced fat in food products. Considerable debate surrounds whether the intake of low-fat or whole fat dairy products are more beneficial for cardiovascular health. Globally, trends show that full-fat dairy consumption has fallen since the 1970s and low-fat dairy consumption has increased [88]. However, studies tend to indicate that whole-fat dairy consumption has a beneficial effect on CVD health and may be more beneficial than low-fat dairy consumption, particularly in relation to inflammatory markers (Table 1). However, low-fat dairy products and whole milk have been associated with lower risk of hypertension in several recent meta-analyses [99,100,101,102]. The Dietary Approaches to Stop Hypertension Trial (DASH) found that low-fat or fat-free dairy foods added significant benefits to vegetables and fruits in lowering blood pressure [103]. Other studies have found dairy consumption in general to be associated with lower blood pressure regardless of the fat content, due to the presence of calcium, vitamin D and other bioactive molecules such as peptides [104,105]. Interestingly, consumption of full-fat dairy as opposed to low-fat dairy products has been beneficially associated with higher vitamin D stores and lower body mass index (BMI), especially in young children [106,107]. 

Researchers from the Observation of Cardiovascular Risk Factors in Luxembourg Survey analysed 1352 adults over 3 months using a semi quantitative food frequency questionnaire, various anthropometric and haemorheological values, while accounting for physical activity and smoking to develop a cardiovascular health score (CHS) that was determined by summing the total number of health metrics at ideal levels. Their resulting data indicates that participants that consumed at least three serving of dairy foods per day had a better CHS than those who consumed less than three servings a day (*p* = 0.04). Additionally, higher intake of full-fat dairy was related to a better CHS (*p* = 0.06) and depending on the fat content of the dairy products, a higher intake of full-fat dairy was associated with a better CHS (*p* = 0.03). Notably, a positive association with total low-fat dairy product consumption was not observed (*p* = 0.22). Furthermore, no association between dairy product consumption and blood pressure, fasting plasma glucose and total cholesterol was observed [22]. However, this study was a cross-sectional study, thus it prevents the establishment of casual inference regarding dairy food intake on CVD health or risk [22,108]. 

It has also been suggested that total and especially full-fat dairy food (*p* < 0.001) intakes are inversely and independently associated with metabolic syndrome in middle-aged and older adults, associations that seem to be mediated by dairy SFA [109]. However, a different study has shown that high intakes of yoghurt and fermented products were cross-sectionally associated with lower odds of presenting with newly diagnosed T2DM and impaired glucose metabolism by 25–40% relative to lower intakes. However, associations of total dairy product intake, full-fat products, skimmed products and Dutch cheese did not reveal similar direction and magnitude for newly diagnosed T2DM or impaired glucose metabolism [110]. These studies suggest that there is a distinct lack of evidence to suggest that full-fat dairy products are associated with a higher risk of CVD, which is in concurrence with several other research groups [10,49,111]. Research also eludes to the fact that low-fat dairy products and full-fat milk consumption are both associated with a reduced risk of hypertension [105] and insulin resistance [112]. Furthermore, fermented dairy products may be more beneficial for cardiovascular health and full-fat dairy consumption in general may have a positive effect on cardiovascular health. Therefore, it is clear that dietary recommendations to avoid full-fat dairy intake are not supported by the literature; further research is required to elucidate the cardioprotective mechanisms of dairy products. 

### 3.4. Limitations to Dairy Research: The Dairy Matrix Effect

Nutritional research traditionally has followed a reductionist, nutrient focused approach that evaluated the effect of a single nutrient group linking one nutrient to one health effect. This may partly explain why discrepancies exist in nutritional research [67]. The classic example of this approach is the link between SFA consumption and CVD. This narrow-minded approach has led to the demonization of foods high in SFA, that have been labelled ‘bad’ for cardiovascular health. In the case of lipids, because of the high heterogeneity of structures and functions, scientists are beginning to realise that different lipids may have differential cardiometabolic effects. There is also increasing evidence that assessing the ‘food matrix’, rather than just a single nutrient may be a more accurate evaluation of the effects of dairy foods on health and needs to be taken into account in food research [90]. This topic has been highlighted and reported by Thorning et al. [113], who concluded that the dairy matrix has specific beneficial effects on cardiometabolic health, body weight and bone health, observations that differ to that of single nutrient constituents. The large numbers of different nutrients combined in a complex physical structure has implications for digestion absorption and metabolism, affecting the overall nutritional properties of the food [113]. They also concluded that different dairy products have the potential to exert different and varying health effects and disease risk markers. Overall, the nutritional value of dairy products should be considered as the biofunctionality of the sum of the nutrients within the dairy matrix structures. A clear example of the dairy matrix effect can be seen in the studies examining the differential effects of cheese and butter on serum cholesterol levels. Another core area of research in lipid studies that needs to be investigated is in estimating frequency, diversity and rate of lipid modifications. Based on previous studies by mass spectrometry, it has been succinctly expressed that lipid modifications do alter bio-functionality but its effect on gastrointestinal digestion has yet to be revealed. Processing treatments induce oxidative and thermal induced modifications that may affect lipid nutritional profile, which in turn encourages formation of advanced lipid end products that accelerate the initiation of CVD. Therefore, sustainable treatment regimens must be deployed to conserve bioactive lipid content. 

## 4. Dairy Products and Cardiometabolic Health

Metabolic syndrome, T2DM, hypertension and obesity are all conditions interconnected with CVD due to similarities in their mechanisms, pathology and systemic inflammation. Systemic inflammation persists in elderly people due to immunosenescence and in those who are obese due to their increased mass of adipose tissue and resulting increase in adipokines. This significantly increases an individual’s risk for endothelial dysfunction and CVD development [4,114]. Metabolic syndrome is a cluster of metabolic risk factors that are associated with increased risk of CVD and T2DM. Metabolic syndrome is typically classified based on an individual exhibiting abnormalities beyond specific parameters of blood pressure, fasting glucose, waist circumference, fasting triglycerides and HDL cholesterol, all of which can worsen with age [115]. Recent research indicates that dairy products may be associated with several beneficial effects on cardiometabolic outcomes.

### 4.1. Dairy Products and Hypertension

Hypertension is one of the leading risk factors for the development of stroke and coronary heart disease. Moreover, novel markers of vascular health include the measuring of arterial stiffness. The health of the walls of blood vessels is a key determinant in cardiovascular disease. Gradual loss in elasticity is a major factor in blood pressure and evidence of ‘hardening’ of the artery as a consequence of atherosclerosis contributes to intra-arterial thrombosis, occlusion and consequent infarction [116]. Arterial stiffness develops with age and can be influenced by diet and lifestyle. Arterial stiffness in an independent predictor of CVD events and mortality [117,118]. Aortic pulse wave velocity and augmentation index are used to determine alterations in vascular function, which are predictive of heart attack and stroke [108,116,118]. Evidence suggests that the intake of milk and dairy products has a positive impact on hypertension [118]. The DASH study [119] has shown reductions in the diastolic and systolic pressures of subjects that consumed low-fat dairy products. This has been seen in later studies that confirmed these relationships by demonstrating a significantly reduced relative risk for hypertension (RR = 0.97) [101]. Another study found a significant reduction of systolic blood pressure in overweight individuals who consumed three servings of low-fat dairy products over 8 weeks [120]. A recent study has shown that hypertensive individuals who consumed ≥2 servings per week of yoghurt were at lower risk of developing CVD [121]. Several aforementioned meta-analyses have found similar associations with dairy intake and blood pressure [100,101]. A recent meta-analyses has found that low-fat dairy products may be associated with the reduced risk of hypertension [102]. Furthermore, Gholami et al. found that total dairy intake demonstrated an inverse association with stroke and CVD [122]. In addition, the Caerphilly Prospective Study demonstrated significant inverse relationships between dairy intake and augmentation index [118]. Mechanistically it is thought that bioactive lipids and peptides may play a role in hypertension reduction [4,123,124]. Therefore, further research is warranted to decipher the mechanisms that govern the observed beneficial health effects of dairy product consumption on hypertension and the risk of stroke. 

### 4.2. Dairy Products and Diabetes

Excess energy intake is associated with the development of obesity and insulin resistance, key metabolic features in the pathophysiology of T2DM. Excess intake of dietary nutrients is an important risk factor for obesity and insulin resistance [125], thus logic would suggest that dairy products being nutrient dense may increase the risk of T2DM. On the contrary, several observational studies have concluded that the consumption of dairy foods is associated with improved insulin resistance [112,126,127,128,129]. Furthermore, a recent meta-analysis of randomised controlled trials concluded that increasing dairy food intakes did not significantly impact cardiometabolic risk factors [130]. Four other meta-analyses that combined data from 4–14 studies spanning 167,000–459,790 subjects are consistent in showing no significant association between milk consumption and the risk of T2DM (RR, ranging from 0.87 to 0.95; 95% CI, ranging from 0.69 to 1.67, depending on whether or not reduced fat or full-fat milk was considered) [131,132,133,134]. A mendelian randomisation study on a Danish cohort of 97,811 individuals demonstrated that milk consumption as assessed observationally or genetically via lactase persistence, was not associated with the risk of T2DM [135]. The large EPIC study of 12,403 cases of T2DM in 8 European countries demonstrated no significant association between total dairy intake and T2DM but combined fermented dairy (cheese, fermented milk and yoghurts) was associated with protection against T2DM with a 12% reduction (*p*_trend_ = 0.02) when comparing extreme quintiles [136]. Particularly in the Norfolk-EPIC cohort, a 24% reduction of risk for low-fat fermented products and a 28% reduction for yoghurt intake (both *p*_trend_ < 0.05) [137]. Furthermore, in a systematic review of the literature, Morio et al. concluded that there is no evidence that dietary saturated fatty acids from varied food sources affect the risk of insulin resistance or T2DM, nor is intake of full-fat dairy products associated with this risk. Moreover, they deduced that the inverse association between dairy consumption and T2DM may be due to other components within the dairy matrix. Therefore, future studies on the effects of dietary saturated fatty acids should take into account the complexity of the food matrix [125]. Mechanistically, it is thought that anti-diabetogenic properties of dairy may be a result of the presence of bioactive lipids such as rumenic and vaccenic acids (Conjugated linoleic acids—CLA), butyric acid and the presence of other biologically active molecules such as phytanic acid, vitamin A and bioactive peptides that may interact with the activation of the peroxisome proliferator-activated receptor-γ (PPAR-γ) [138]. The totality of the evidence indicates that dairy consumption, in particular fermented dairy products are associated with a reduced risk of T2DM.

### 4.3. Dairy Products and Obesity

Obesity is an important risk factor for the development of CVD and other chronic diseases. The rate of obesity among children in developed countries such as Ireland and the United States continues to be a major concern due to the increased risk of CVD and T2DM [139,140]. Dairy foods characterised by their high SFA content and calorie content, pose a potential risk for weight gain and obesity. However, research thus far has led to conflicts and contradictions concerning this notion. Several cross-sectional studies have shown an inverse relationship between body mass index (BMI) or adiposity and dairy intake in children [139,141,142], however this is not consistent between studies [143]. Some studies indicate a slight weight gain when eating full-fat dairy products [105]. A systematic review of 10 cohort studies demonstrated an inverse association between yoghurt consumption and the risk of overweight or obesity but this was not uniformly consistent or statistically significant [144]. A further study has shown that dairy product consumption associated with reduced risk of obesity in Korean women but not in men [23]. Although many studies demonstrate an inverse association between dairy products and the risk of being overweight or developing obesity, further studies are imperative to fully understand these complex associations and provide accurate dietary guidelines to consumers for weight management. 

## 5. Anti-Inflammatory Properties of Dairy Products

Low-grade inflammation is the key biological phenomenon underpinning the development and progression of CVD, metabolic syndrome and T2DM. The initiation and resolution of the inflammatory response involves the complex and coordinated expression of inflammatory compounds, which induce a myriad of physiological processes, ranging from local vascular response to systemic responses affecting the whole organism [53]. During atherosclerosis, circulating inflammatory mediators actively contribute to vascular and atheromatous change [53,145]. As thoroughly reviewed by Da Silva and Rudkowska [145], there are multiple studies that have examined the role of dairy components on various cell lines and found that generally these components have an inverse association with inflammation. In particular, long-chain SFA such as palmitic (C16:0) and stearic (C18:0) may exhibit pro-inflammatory effects. Although these fatty acids are both found in abundance in dairy products, as evidenced by the lack of association between dairy products and CVD, it is suggested that the deleterious effects by these SFA in milk is offset by other dairy components [145]. Other SFA such as lauric acid (C12:0) may have a neutral or anti-inflammatory effects, however further research is required in humans [145]. 

Platelet-activating factor (PAF) is a potent pro-inflammatory phospholipid mediator implicated in the initiation and progression of atherosclerosis [146]. PAF and PAF-like molecules act through their binding to a unique G-protein coupled seven transmembrane receptors (PAF-receptor), which subsequently triggers multiple intracellular signalling pathways, depending on the target cell and PAF levels in the tissue and blood [53,147]. PAF in general plays a central role in various physiological processes, such as mediation of the normal inflammatory responses, regulation of blood pressure and regulation of coagulation responses [53]. Potential therapeutic approaches to the pro-inflammatory actions of PAF focus on the PAF/PAF-receptor interactions, thus inhibiting the exacerbation of the complex PAF-induced inflammatory response and pathways through competitive and non-competitive displacement of PAF from the PAF-receptor [53]. A number of PAF inhibitors and/or antagonists have been identified in the polar lipid fractions of numerous food types, including dairy products [53]. It seems that bovine, ovine and caprine dairy products possess polar lipids with potent anti-inflammatory activities as demonstrated in a series of in vitro experiments on washed rabbit platelets [4,148]. Research has shown that as milk is fermented to yoghurt and then to cheese, the bioactivity of the PAF inhibitors seems to increase [4]. This indicates that the processes of fermentation and lipolysis play a key role in altering the bioactivity of the polar lipid fractions of milk and this bioactivity increases the further fermentation proceeds [4,53]. These effects have been attributed to microorganisms such as *Lactobacillus delbrueckii* ssp. *bulgaricus* and *Streptococcus thermophilus*. Research also indicates that polar lipids of caprine and ovine milk and dairy products possess greater bioactivity than those of bovine milk and dairy products [148,149,150,151,152].

Many reviews have highlighted that dairy products are associated with positive effects on cardiovascular health, particularly in epidemiological studies. It is clear from Table 1 that several intervention studies, crossover studies, cross-sectional studies and randomised controlled trials (RCT) indicate that dairy consumption may be cardioprotective due to lower levels of inflammatory markers including TNF-α, IL-6, IL-13, MCP-1 and VCAM-1. Table 1 indicates that dairy products are associated with a neutral or positive effect on inflammatory markers in both healthy and diseased individuals in various forms of human trials. However, the mechanisms underlying the observed inverse associations between the intake of specific dairy products and inflammation remain elusive. Some mechanisms have been suggested for specific fatty acids such as CLA and various bioactive proteins [4,153]. As highlighted in Table 1, many studies that have evaluated the effects of dairy product consumption on inflammatory markers tend to focus on low-fat dairy products, a point that has been previously highlighted by Lordan and Zabetakis [4]. The study of only low-fat dairy product consumption may have several limitations because of the reduced intake of the anti-inflammatory lipids of dairy products. Therefore, the observed neutral effects of dairy product intake on inflammatory markers in Table 1, may be due to the assessment of only low-fat dairy products in some studies. The prominent focus on the study of low-fat dairy may be due to the negative perceptions associated with full-fat dairy products in society, thus low-fat dairy products may be consumed more in the populations assessed. In addition, there are also indications that the observed effects of dairy intake on inflammatory markers may be dose dependent [154]. Notably in Table 1, fermented dairy products tend to reduce inflammatory markers more than non-fermented products, which mechanistically may explain the observed greater health benefits of fermented dairy consumption versus non-fermented dairy products. Further studies are required to assess the effects of dairy product consumption and dairy lipid constituents on inflammatory markers and cardiovascular health [4].

## 6. Trans Fatty Acids

*Trans*-fatty acids (TFA) have previously been associated with an increased risk of CVD [173]. TFA affect many CVD risk factors by increasing: LDL; lipoprotein(a); serum triglycerides; LDL particle number; shifting LDL subclasses to more atherogenic small dense LDL; increasing inflammation; and reducing HDL levels [174,175,176]. Thus, many dietary guidelines recommend limiting dietary TFA intake to less than 1% of energy intake [28,177,178] and some countries such as Austria, Hungary, Iceland, Latvia, Norway and Denmark have introduced legal bans that limit the percentage of artificial TFA in oils and fats. In Denmark that limit is just 2% (2 g per 100 g) [179]. Other countries including Lithuania and Sweden are close to adopting similar legislation. Dietary intake of TFA is characterised by both industrial-TFA and ruminant TFA. In the margarine and cooking oil industries, during the process of fat hardening, partial hydrogenation or deodorisation of vegetable oils can lead to the production of artificial industrial TFA. In processed fats, elaidic acids (C18:1t9) are the most prominent TFA, followed by *trans*-vaccenic acid [180,181]. In dairy products and ruminant fat, some *trans*-isomers are produced naturally in small quantities by microorganisms in the rumen of ruminant animals, which occurs due to the partial hydrogenation of *cis*-fatty acids, primarily linoleic acid and α-linolenic acid. The most abundant TFA is *trans*-vaccenic acid (C18:1t11) [180,182]. Other biologically important TFA include conjugated linoleic acids (CLA) such as rumenic acid (C18:2c9t11) and (C18:2t10,c12), which are associated with a number of health benefits [4]. Notably except for CLA, all TFA in industrially produced fat are also found in ruminant fat but the amounts of the individual TFA differ significantly strongly between both types of fat [180]. 

Dietary intake industrial TFA increased after a surge in the production of industrial fats between the 1960s and 1980s, in response to public health recommendations to ironically replace animal products and tropical oils, both high in SFA [183]. However, as research advances generalising fatty acids by the degree of unsaturation or the configuration of double bonds alone is unlikely to predict biological responses. Thus, emerging evidence suggests that TFA from ruminant sources only may not be as detrimental to health as previously thought [173,184], in fact some may even have cardioprotective effects [176]. Ruminant *trans*-fatty acids constitute a typically 2–5% of the fat in dairy products [185]. Generally, ruminant TFA accounts for 2–9% of fatty acid intake [186]. In addition, the TRANSFAIR Study estimates that as much as half of all *trans*-fats consumed are ruminant TFA in specialty diets, such as the Mediterranean diet [187], which is associated with positive cardiovascular health benefits [188]. A study was conducted feeding either industrial TFA or ruminant TFA to LDL receptor deficient mice (LDLr−/−); mice fed a diet of cholesterol supplemented with industrial TFA (elaidic acid) stimulated atherosclerosis and plaque formation, whereas plaque formation was reduced in mice that were fed the same cholesterol rich diet but consumed butter rich in vaccenic TFA (18:1t11) instead of industrial TFA [186]. This may indicate a protective effect for ruminant vaccenic TFA against atherosclerosis. A case control study in Costa Rica found that an adequate concentration of c9,t11-CLA in adipose tissue was associated with a lower risk of myocardial infarction (MI; highest versus lowest quintile; OR = 0.51; 95% CI = 0.36–0.71; *p* < 0.0001) and that dairy intake was not associated with risk of MI, despite a strong risk associated with saturated fat intake [189]. A meta-analysis of cohort studies found no association between ruminant TFA intake and CVD risk (RR, 0.92 (95% CI, 0.76–1.11); *p* = 0.36), however suggested further research for confirmation. Further studies indicate that c9, t11-CLA exhibit potent anti-inflammatory effects against IL-6 and TNF-α expression, as well as adiponectin secretion in 3T3-L1 adipocytes [190,191]. A recent study has also shown that high dairy fat intake was not associated with incident coronary heart disease but was associated with reduced risk of HF, largely because of the inverse effect of the presence of serum CLA (measured as a % of total fatty acids), which is elaborated further in Table 1 [172]. Furthermore, as reviewed by Lordan and Zabetakis [4], CLA enriched dairy products has a neutral or positive effect on circulating inflammatory markers and lipid profiles of healthy and diseased participants.

There is also evidence to suggest that circulating *trans*-palmitoleic acid (C16:1t9), which occurs in both dairy fat and partially hydrogenated oils, is associated with lower atherogenic dyslipidaemia, insulin resistance and the incident of diabetes, which may explain previously observed metabolic benefits of dairy consumption [192]. However, controversy remains, thus more research is required to differentiate between artificial and ruminant TFA on CVD risk factors [193]. It is clear that TFA of industrial origin are associated with an increased risk of CVD. Putative evidence suggests that TFA of ruminant origin may be associated with beneficial effects against CVD, however further research with a focus on dairy products is required to confirm these observations.

## 7. Fermented Dairy Products and Cardiovascular Health

Fermented milk beverage consumption is on the rise due to consumers’ perception of its healthy effects, widely disseminated by increasing numbers of studies describing the importance of the different nutrients and bioactive compounds [194]. Fermented dairy products include various yoghurts, cheeses and fermented milk products such as *kefir*. Fermented dairy products are synonymous with the delivery of probiotics, which are a microorganisms that are alive when they arrive to the gut and have the potential for therapeutic and preventative health benefits upon consumption by improving host intestinal microbiota [195]. Fermented dairy products tend to possess more health benefits than fluid milk upon consumption [148]. Increased consumption of fermented dairy foods is associated with reduced LDL cholesterol [10], reduced hypertension risk [123] and CVD risk [17], there is also a suggestion that there may be a dose response [196]. Although these patterns are observed in several studies (Table 1), dairy food intake is associated with many confounders that are generally associated with better health outcomes such higher educational levels and socioeconomic status [31,197]. Furthermore, children who consume >60 g of yoghurt a day have a higher overall diet quality, nutrient intake, lower pulse pressure (4–10 years old) and lower HbA1c concentrations (11–18 years old) indicating favourable cardiometabolic health [198].

A recent study comparing fermented and non-fermented dairy products with all-cause mortality in a Swedish cohort found that there was a 32% increased hazard (HR: 1.32; 95% CI: 1.18–1.48) in high consumers of non-fermented milk (≥2.5 times/day), when compared to consumers of milk (≤1 time/week), Whereas butter was 11% (HR: 1.11; 95% CI: 1.07–1.21). All non-fermented milk-fat types were independently associated with increased HRs but were lower in consumers of medium and low-fat milk, when compared with full-fat milk. Fermented milk intake (HR: 0.90; 95% CI: 0.86–0.94) and cheese intake (HR: 0.93; 95% CI: 0.91–0.96) were negatively associated with mortality [199]. Meta-analyses have shown that fermented dairy products can have an inverse association with T2DM [132,136] and cheese consumption was not associated with an increased risk of all-cause mortality [12,200]. In a crossover-controlled study, yoghurt consumption has also been shown to increase HDL levels in 29 hypocholesterolaemic women The HDL concentration increased significantly by 0.3 mmol/L (*p* = 0.002). The ratio of LDL/HDL cholesterol desirably decreased from 3.24 to 2.48 (*p* = 0.001) [201]. A recent meta-analysis supports the observed associations that fermented dairy product consumption had a positive or neutral effect on CVD risk [102]. In particular, fermented dairy intake was associated with a reduced risk of stroke and T2DM. Other studies have found that fermented dairy products have mostly positive or neutral effects on fasting plasma glucose levels [202], however one study has shown that fermented milk lowers fasting plasma glucose levels in patients with T2DM [203].

As current dietary guidelines generally place an emphasis on the reduction of SFA intake, it would be expected that cheese consumption would be associated with an increased risk of CVD. Cheese provides a high intake of SFA and cholesterol and is also a major source of calcium and protein [204]. Epidemiological evidence indicates that cheese consumption may be less atherogenic than previously assumed. Observational studies have failed to identify a significant association between high cheese or dairy fat intake and coronary heart disease [205,206,207]. Cheese consumption has been associated with a significantly reduced risk of stroke and CHD [208]. Similar effects have been observed in yoghurts, which are a diverse complex nutrient-rich matrix that have been associated with lower incident risk of CVD [209], diabetes [20] and metabolic syndrome, particularly when consumed with fruit [144,210]. Mechanistically these effects may be due to the presence of bioactive lipids and peptides with anti-inflammatory properties [4], and/or the observed effects of high calcium intake from cheese, which may lower SFA intake, reducing the risk of high cholesterol levels. 

Several epidemiological studies have found that populations that traditionally consume high amounts of fat have low incidences of CVD and mortality, which was first defined as the French paradox by Renaud and De Lorgeril [211]. It is thought that high consumption of cheese and wine in the French diet may be responsible for these effects. In particular, bioactive molecules in dairy products can stimulate intestinal alkaline phosphatase, a potent endogenous anti-inflammatory enzyme [212]. It is thought that bioactive lipids and peptides and biomolecules from the cheese moulds may be a number of cardioprotective properties, including angiotensin-I converting enzyme (ACE) inhibitors and molecules regulating haemorheological functions, blood coagulation and blood pressure [212]. In particular, cheeses that are moulded such as Camembert and Gorgonzola may have positive effects for cardiovascular health; especially Roquefort, which is a blue cheese is particularly cardioprotective due to the presence of bioactive molecules such as andrastins A–D and roquefortine [213]. Fermented dairy products may also induce their cardioprotective effects due to the intake of bacterial metabolites and probiotics. Probiotics arrive to the gastrointestinal tract alive, where they can exert their effects directly. Probiotic intake due to supplementation or consumption of fermented dairy products has been associated with potential cardiovascular health benefits, including positive effects on blood pressure and hyperlipidaemia and they may exert anti-inflammatory activities [214]. The mechanistic explanations surrounding the positive effects of fermented dairy product consumption remain elusive. Further studies may help to elucidate the intriguing cardioprotective effects of moulded cheeses that may help to elucidate the mechanisms that are responsible for the French paradox. 

Fermented dairy products are an excellent source of vitamin K. Vitamin K is essential to blood clotting and coagulation as it is an enzyme cofactor for γ-carboxylation of peptide-bound glutamate residues [215]. However, vitamin K is also involved in the regulation of bone and soft tissue calcification, cell growth and proliferation, cognition, inflammation and oxidative processes [216]. In particular, fermented dairy products contain menaquinones—also known as vitamin K_2_—a collection of isoprenologues mostly originating form bacterial synthesis [217]. Vitamin K_2_ includes a range of vitamin K isoforms that differ structurally from vitamin K_1_ also known as phylloquinones, which are mainly found in leafy green vegetables. Vitamin K_2_ is the most biologically active form of vitamin K and it has a longer half-life than vitamin K_1_ [36,218,219]. A growing body of evidence suggests that vitamin K has beneficial effects on cardiovascular health [220,221,222,223]. Observational studies have demonstrated that low vitamin K status plays a potential role in CVD development, particularly in high-risk populations and individuals suffering chronic kidney diseases [36,224]. In comparison to other foods, fermented dairy and other animal products have a high content of vitamin K_2_. Fermented dairy products, in particular cheese, yoghurt and *kefir* contain vitamin K_2_ [219,225], thus consumption of fermented dairy products may be beneficial for human cardiovascular health. Randomised trials have provided some evidence to support the beneficial effects of vitamin K_2_ on bone health and some intervention trials that assessed vitamin K in conjunction with vitamin D have demonstrated positive results on cardiovascular-related outcomes [36]. However, randomised trials have not yet examined vitamin K_2_ intake in relation to cardiovascular outcomes [36,217].

**Figure 1:**
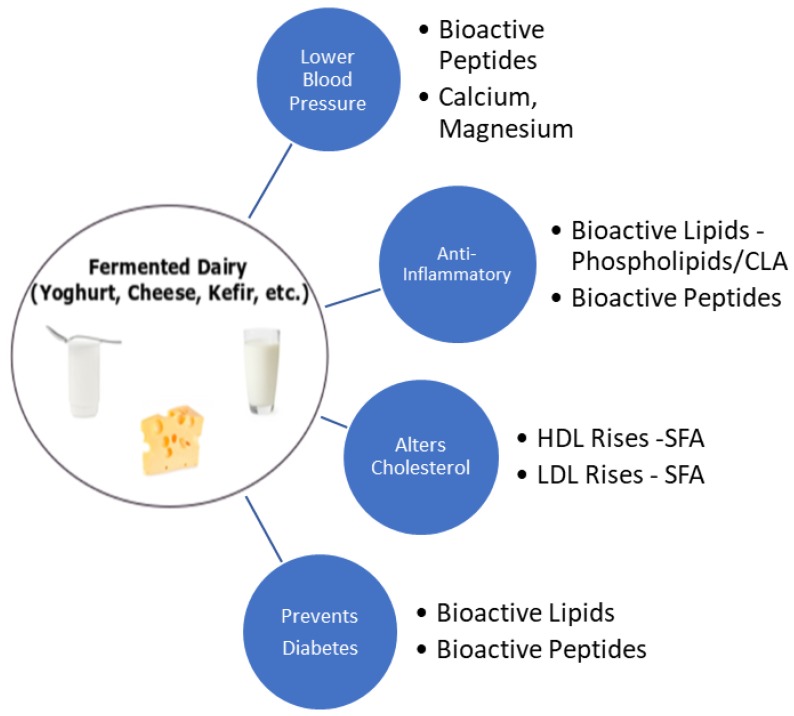
Effects of dairy product consumption on cardiometabolic risk factors and cardiovascular health. CLA: conjugated linoleic acid; HDL: high-density lipoprotein cholesterol; LDL: low-density lipoprotein cholesterol; SFA: saturated fatty acid.

In summary, fermented dairy products have positive effects on cardiovascular health (Figure 1) and may be even more beneficial than non-fermented dairy products. However, none of the putative bioactive compounds in dairy foods such as proteins, lipids, phospholipids, vitamin D, vitamin K, or probiotic bacteria has consistently explained the benefits of dairy intake on cardiovascular health. The process of fermentation leads to the structural change of lipids and proteins in cheese and yoghurt, which may be responsible for some of the observed effects [4,226]. Several bioactive peptides and phospholipids present in fermented dairy products have protective effects, including the anti-inflammatory effects of phospholipids and the antioxidant properties of plasmalogens. The structure of lipids and phospholipids in the milk fat globule membrane (MFGM) may also play a role in modulating plasma cholesterol levels, further supporting the evidence surrounding the food-matrix effect [226,227]. How these different constituents and properties influence cardiovascular risk factors is poorly understood. Mechanistic studies are key to identifying the missing links that explain the positive cardiovascular health benefits of consuming fermented dairy products.

## 8. Functional Alternative Dairy Foods and Consumer Trends

Increasing interest in the relationship between diet, health and well-being is likely to drive a growth in demand for products that have positive impacts on health. Consumer research indicates, that the functional food markets are growing worldwide and that there is a greater demand for functional foods against specific diseases [120]. Dairy products lead the charge in this field in terms of the creation of dairy products low in fat, or containing probiotics, bioactive peptides, altered lipid profiles, cholesterol lowering capabilities and vitamin, protein and mineral fortification [228,229,230].

### 8.1. Cholesterol-Lowering Dairy products

Several food producers have taken it upon themselves to introduce dairy products to the market that target hypercholesterolemia, which is caused by high SFA intake. Phytosterols and their saturated form phytostanols are a group of steroid alcohols naturally present in fruits, legumes, nuts, seeds, vegetables and vegetable oils [231,232]. These molecules have many applications and are generally found as food additives or ingredients in ‘cholesterol-lowering’ dairy products. The inclusion of stanols/sterols as an ingredient in foods is recognised as safe by the European Food Safety Authority [233]. The function of plant stanol esters is to partly block cholesterol absorption in the digestive tract and thereby reduce total cholesterol and LDL cholesterol levels. Structurally, they are similar to cholesterol but their absorption rate is much lower. Foods with added plant stanol esters may provide the consumer an opportunity for cardiovascular disease prevention [230,234]. As consumer awareness increased, the number of products containing plant sterols or plant stanols and their esters has increased. In addition, milk-based beverages containing phytosterols have gained a distinct place among these type of functional foods, as phytosterols do not readily dissolve in water based products and so low-fat versions are not as common [235]. Some studies have seen a benefit for the consumption of phytosterols to lower serum cholesterol levels. One study showed that the consumption of 1.7 g/day of phytosterols by hypercholesterolaemic men had a lowering effect on serum cholesterol levels [236]. Another study found daily consumption of low-fat milk containing 1.6 g phytosterols was effective in reducing LDL levels by 8% after 6 weeks in 194 hypercholesterolaemic individuals [237]. In a recent randomised double-blind crossover, placebo-controlled study in moderately hypercholesterolaemic subjects (*n* = 40) aged between 20 and 50 years old, who consumed a yoghurt drink with 4 g of plant stanol esters (Benecol**^®^**) compared to a normal yoghurt drink caused a statistically significant decrease in total cholesterol and low density lipoprotein cholesterol by 7.2% and 10.3% [230]. Similar products to Benecol**^®^** exist on the market such as Flora ProActiv yoghurt drink, which claims to be ‘clinically proven to reduce cholesterol’ [233]. Although studies show that these products may lower serum cholesterol levels and thus possibly lower cardiovascular risk, as previously discussed, lowering serum cholesterol levels alone is not enough to prevent CVD development. Therefore, long term studies are required to assess the effects of long term consumption of phytosterols on cardiovascular health. 

### 8.2. Plant Based Milk Alternatives

Plant-based alternatives to dairy products have been produced to satisfy consumer needs and to accommodate an increased uptake of vegan, ‘clean eating’ lifestyles and lactose intolerance. In fact, in many countries fluid milk consumption has decreased, whereas the consumption of plant-based alternatives is on the rise. Almond milk has surpassed soy milk as the main alternative beverage consumed in the United States. Plant milk substitutes are suspensions of dissolved and disintegrated plant material, resembling cow’s milk in appearance [238]. These ‘milk’ products include soy, almond, rice, coconut, flax or hemps milk. Each product is identified as a satisfying alternative for consumers who desire a milk-like consistency for cereal or other food combinations [239]. These products are generally perceived as “healthier” by consumers and each product is marketed to promote a different health benefit, as consumers are trending away from animal-based products over concerns about lactose, fat and cholesterol [239]. The nutritional properties of these products are highly variable and depend on the plant source, processing and fortification. However, these products are not nutritionally similar to dairy products, often inferior and as such they are not considered a part of the dairy food group in nutritional guidelines [27,240]. 

Some products have extremely low calcium and protein contents and so consumer awareness is imperative, when considering the use of plant-based substitutes for cow’s milk in the diet [240]. Many of these beverages also contain high amounts of sugar, which may have consequences to frequent consumers [240]. In general, little research has been done to assess the health benefits of these products, however as soy milk has been popular for over 25 years more research exists. Although not completely established, it is thought that soy protein may have hypocholesterolaemic effects [241], reduce blood pressure [242] and various other risk factors, however the data remains inconsistent [243]. Some studies indicate that soy products may possess anti-inflammatory properties, however a recent study on mice has shown that bovine milk possessed anti-inflammatory properties, whereas soy bean was pro-inflammatory in an mouse model of obesity [244]. In addition, it is not yet known what effect long-term consumption of plant-based alternatives will have on cardiometabolic health. It is generally assumed that as they are plant-based products they will have a positive impact on cardiovascular health. However, apart from soy products, there is a distinct lack of research and epidemiological evidence to substantiate any health claims in relation to plant-based alternative beverages and their cardiometabolic health effects. Therefore, plant-based beverages have not been accepted by everyone. In December 2016, producers of plant-based beverages were challenged by members of the United States Congress and the dairy industry; A request was made to the American Food and Drug Administration to demand that producers refrains from using the words ‘milk’, ‘yoghurt’ or ‘cheese’ to describe their plant-based products. Concerns were voiced about their nutritional quality and it was claimed that the producers were misleading their customers [245]. A bill was introduced to the House of Representatives and was referred to the Subcommittee on Health, which has yet to carry out any further action. 

### 8.3. Alternatives to Bovine Milk: Caprine and Ovine Milk

Bovine milk is consumed globally and accounts for 85% of the total global milk production, followed by buffalo (11%), goat (2.3%), sheep (1.4%) and camel milk (0.2%) [246]. Interestingly, despite the dominance of bovine milk production, on a global basis, more people consume goat milk than milk from any other single species [247]. This demand is also due to an increased trend of health conscious consumers and an increased purchase of caprine dairy products due to allergy issues related to bovine products [248]. The production of ovine and caprine dairy products is prominent in the Mediterranean basin and the Middle East. Outside of these distinct regions, sheep and goat milk products are considered a delicacy and their consumption is not common particularly in countries like Ireland and the UK where consumption of these products is not widespread [148]. Neither caprine or ovine dairy products have been extensively studied for their effect on cardiometabolic health, despite their high levels of consumption in developing countries. The lipid fraction of caprine milk is significantly different to bovine milk and is a much-overlooked component. First of all, the lipid in both caprine and ovine milk is present in fat globules, which in ovine products are less than 3.5 µM, among ruminants sheep milk fat globules are the smallest [249]. This is advantageous as both caprine and ovine milk have increased digestibility, thus the milk lipid is metabolised more efficiently in humans. This may be beneficial for the uptake of bioactive lipids present in the milk fat of caprine and ovine milk products [4,148]. 

Caprine milk exceeds bovine milk in MUFA, PUFA and medium chain triglycerides (C6–C10, length of the carbon chain), which all are known to be beneficial for human health, especially for cardiovascular conditions [250]. Caprine milk is also high in vitamins A, B1 and B12 as well as calcium and phosphorus content when compared to bovine and ovine milk [249]. Caprine milk fat content is approximately 3.8%, ovine milk fat is 7.9%, whereas bovine milk is approximately 3.6% [251]. Both caprine and ovine milk contain bioactive lipids such as CLA, which account for 2–4%, of total fatty acids esterified in phospholipids [252]. These lipids are associated with a number of anti-inflammatory properties that may have positive effects on cardiovascular health [4] Studies using ovine cheese have established that there is a reduction of inflammatory markers, platelet aggregation [156], modulation of plasma lipid profiles and a reduction of endocannabinoid biosynthesis upon ovine cheese consumption [253]. Similarly, caprine milk, yoghurt and cheese has the potential to reduce platelet aggregation in humans [150,152]. It is also suggested that the polar lipid (phospholipid and sphingolipid) content of caprine and ovine dairy products may be responsible for some of the antithrombotic effects observed [4,53]. 

Ovine milk and dairy products have been associated with positive effects on cardiovascular health [148]. The world’s largest producer of sheep milk is China (12.2%) and the leading producer in Europe is Greece (8.7%), followed by Romania (7.2%) and Italy (6.1%) [254], in stark contrast to Ireland where ovine dairy farms are rare. Sheep’s milk is mainly used for the production of fine cheese varieties, yoghurt and whey cheeses [255]. The high levels of protein, fat and calcium by casein unit make it an excellent matrix for cheese production [256]. The nutritional value of sheep milk is higher than that of either bovine or caprine milk, with higher levels of proteins, lipids, minerals and vitamins essential to human health [246,254]. Ovine milk is high in caproic, caprylic and capric acid. The most predominant fatty acids in sheep milk and yoghurt are oleic acid (C18:1n9), followed by palmitic acid (C16:0) and myristic acid (C14:0), respectively [246]. Human diets high in oleic acid are reported to decrease the level of LDL cholesterol, whereas HDL cholesterol levels are not significantly affected [148,257]. A recent study has shown that yoghurt intake, from either ewe’s or cow’s milk, at levels of consumption compatible with a varied diet, neither decreases nor increases plasma lipoprotein cholesterol levels in apparently healthy individuals [194]. These neutral effects on serum cholesterol levels and the positive anti-inflammatory and antithrombotic effects that have been observed in humans indicate that ovine dairy products, in particular their lipid fraction may be cardioprotective upon consumption. In addition to their bioactive lipid fraction, dairy products derived from ovine milk have angiotensin-converting enzyme (ACE) inhibitor peptides that also have positive effects on cardiovascular health [258,259]. Therefore, fermented caprine and ovine dairy products may possess potent cardiovascular health effects that warrant further investigation.

### 8.4. Functional Foods—Kefir

Consumer trends for functional foods has led to the increase of various yoghurt drinks on the market. However, *kefir* milk has gained considerable attention for its putative health benefits. When milk is inoculated with *kefir* grains they produce acidified fermented milk that is slightly carbonated and contains a small amount of alcohol. The aforementioned *kefir* grains are microbially derived protein and polysaccharide matrices that contain a community of bacterial and fungal species that are essential to *kefir* fermentation [260]. When fermenting, lactic acid, bioactive peptides, exopolysaccharides, antibiotics and bacteriocins are produced [225,261] and the fatty acid composition is altered [262]. The microorganisms in *kefir* have probiotic potential and may have a positive impact on gut health. However, the microbial composition of *kefir* is highly diverse and it is not known what health benefits can be attributed to the actions of specific microbes that are present in the matrix [263]. *Kefir* has been associated with a number of positive effects on blood lipid profiles in animal models [264,265] which has been reviewed in great detail by Rosa et al. [225]. However, in mildly hypercholesterolaemic men who consumed *kefir* as part of their diet for 4 weeks there was no significant change to total serum cholesterol, LDL cholesterol, HDL cholesterol, or triglyceride concentrations [266]. Another study examined the effect of *kefir* on the glucose and lipid profile in patients with T2DM. They demonstrated that *Kefir* milk decreased fasting glucose and HbA1C levels and can be useful in the prevention of T2DM [267]. Other animal models have also demonstrated that *kefir* milk may possess a number of anti-inflammatory properties [225] but these effects have yet to be demonstrated in humans. Although discrepancies may exist between animal and human studies, it may be in large part due to the fact that different *kefir* grains were used for each of these studies and the study timeline were different [263]. It seems that *kefir* milk is highly unexplored and although there are a resounding number of studies that claim various health benefits, it remains to be seen what effect *kefir* milk has on human cardiovascular health.

## 9. Conclusion: Dairy Fats and Cardiovascular Diseases, Do We Really Need to Be Concerned?

The role that dairy products play in human health has been contested for decades. Generally the literature agrees that dairy products are neutral or beneficial to human health as evidenced by several meta-analyses and randomised, controlled trials [4]. However, do we really need to be concerned about whole fat dairy product consumption? Or should dietary recommendations continue to support the consumption of low-fat or non-fat dairy products, while limiting the intake of full-fat milk and dairy? Previous research indicated that reducing serum cholesterol levels would lower cardiovascular risk, however, CVD are a multifaceted disease, which requires a multifaceted approach to primary prevention. It is clear the reductionist approach to dietary research and the formation of dietary guidelines is obsolete in the face of new evidence suggesting that systemic inflammation is the key underlying biochemical phenomenon at the centre of atherosclerosis and the onset of major cardiovascular events [4,53]. 

Apart from genetic and environmental influences, maladaptive diet and lifestyle are central to the development of CVD and are a key modifiable risk factor for its prevention [53]. Despite the previous concerns about dairy product consumption due to the SFA content, it has been shown that not all SFA are created equal and that the presence of specific fatty acids (C14:0,C15:0, C17:0, CLA and *trans*-palmitoleic) in circulation are associated with a lower incidence of several cardiometabolic diseases, however some may simply be markers of dairy intake [268]. Subsequent research indicates that the dairy food matrix also plays a major role in nutritional research. Consequently, the predicted health benefits of some foods based on their individual nutrient composition does not always exhibit the predicted effect in clinical research. Further human studies may discern the mechanisms surrounding fatty acids and their individual effects on cardiometabolic health. 

Several meta-analyses point to the resounding conclusion that, although dairy products contain a high SFA content, their consumption induces a positive or neutral effect on human cardiovascular health [16,17,269]. In addition, consumption of full-fat dairy products contributes to higher intakes of significant nutrients, in particular vitamin D and vitamin K. Considering current scientific evidence, after years of controversy the negative image of milk fat is weakening. Therefore, consumers can continue to moderately consume full-fat dairy products as part of a healthy and balanced lifestyle, however fermented dairy products would be preferential for optimum nutrient intake and potential cardiovascular health benefits. The authors suggest that less emphasis is needed on the impact of milk and dairy product consumption on serum cholesterol levels but more emphasis should be placed on inflammatory biomarkers to elucidate the cardioprotective mechanisms of dairy products.

## Figures and Tables

**Table 1:** Summary of the findings of observational studies investigating the consumption of dairy products and their derivatives on inflammatory markers related to cardiovascular diseases, obesity and metabolic syndrome in healthy and diseased individuals.

Author, Year	Country	Study Design	Study Focus	Outcome	Conclusion
Thompson, 2005 [155]	USA	Dietary intervention	The effects of high-dairy and high-fibre consumption on weight loss in 90 obese subjects was assessed	CRP was reduced by 0.8 mg/L from baseline (*p <* 0.0001), however there was no significant difference between the dairy diet and the others tested	An insignificant reduction of CRP was observed following dairy consumption in obese participants
Sofi, 2010 [156]	Italy	Dietary intervention	Effect of pecorino cheese naturally enriched with *cis*-9, *trans*-11 CLA on inflammatory markers in 10 healthy participants	Reduction in arachidonic acid-induced platelet aggregation (pre: 87.8 ± 1.76% vs. post: 77.7 ± 3.56%; *p* = 0.04), improvement of erythrocyte filtration rate and a reduction of TNF-α (40.1%), IL-6 (43.2%) and IL-8 (36.5%)	Dietary short-term intake of pecorino cheese rich in *cis*-9, *trans*-11 CLA caused favourable biochemical changes of inflammatory and atherosclerotic markers
Rosado, 2011 [157]	Mexico	Dietary intervention	Effect of adding low-fat milk on anthropometrics, body composition, CRP etc. in energy restricted diets in 139 women	Change in CRP after low-fat milk was 0.2 mg/L (95% CI 1.1–1.6)	Dairy intake had no significant effect on CRP concentrations
Stancliffe, 2011 [154]	USA	Dietary intervention	Effects of an adequate full-fat dairy diet versus low-dairy (both mainly milk and yoghurt) intake on inflammatory markers in 40 overweight individuals with metabolic syndrome over a 12-week period versus a low-fat control	After 7 days, the adequate full-fat dairy diet decreased plasma malondialdehyde and oxidised LDL (35% and 11% respectively, *p* < 0.01), TNF-α decreased by 35% (*p* < 0.05), which further decreased by week 12. By week 12, decreases in IL-6 (21%, *p* < 0.02) and MCP-1 (24%, *p* < 0.05) were observed. Low-dairy intake exerted no effects on oxidative or inflammatory markers	An increase in dairy intake attenuates oxidative and inflammatory stress in metabolic syndrome
Nestel, 2012 [158]	Australia	Dietary intervention	Assessing the effects of low-fat or fermented dairy product intake on inflammation and atherogenesis on 13 overweight participants, using 5-single meal tests	No significant changes in the levels of inflammatory biomarkers (CRP, IL-6, IL-13, TNF-α, VCAM-1 and others) were observed	Authors could not confirm the reported increments in inflammation after high fat meals
Esmaillzadeh, 2010 [159]	Iran	Cross-sectional	Assessing the effect of dairy products on inflammatory markers in 486 women	Low-fat dairy was inversely associated with CRP (β = −0.04), IL-6 (β = −0.02) and VCAM-1 (β = −0.06); high fat dairy was positively associated with log-transformed values of serum amyloid A (β = 0.08) and VCAM-1 (β = 0.05)	Evidence suggests there is an independent relationship between dairy consumption and some markers of inflammation and endothelial dysfunction
Panagiotakis, 2010 [160]	Greece	Cross-sectional	The evaluation of effects of dairy product consumption on levels of inflammatory markers in blood samples from fasting adults with no evidence of previous chronic inflammatory disease	Levels of inflammatory markers such as CRP, IL-6 and TNF-α were 29, 9 and 20% lower, respectively (*p* = 0.01), in people who consumed more than 14 servings of dairy per week compared with those who had fewer than 8 servings per week (*p* = 0.05)	This inverse association between dairy consumption and levels of inflammatory markers in healthy adults indicates that dairy products may be protective against chronic inflammatory diseases
Wang, 2011 [161]	USA	Cross-sectional	305 adolescents were tested for serum phospholipid fatty acid markers of dairy intake (C15:0 & C17:0), which were linked to biomarkers of inflammation by generalised linear regression analyses adjusted for age, gender, race, tanner score, total energy intake and physical activity	Phospholipid dairy fatty acids, elevated by dairy consumption, were inversely associated with CRP, 8-iso-PGF2α and urinary 15-keto-dihydro-PGF2α in overweight but not in normal weight adolescents (all *p*_interaction_ < 0.05). However, higher PL dairy fatty acid levels were associated with lower IL-6 among all adolescents. Adjustment for dietary intake of calcium, vitamin D, protein, total flavonoids and ω-3 fatty acids did not alter the findings	Dairy-specific saturated fats C15:0 and 17:0 fatty acids, may contribute to the potential health benefits of dairy products, especially for overweight adolescents
Gadotti, 2017 [162]	Brazil	Cross-sectional	To assess the effect of dairy consumption and plasma inflammatory markers in 259 participants. Subjects were assigned groups depending on inflammatory status and multiple logistic regression tests were conducted to estimate the odds ratio (OR) for the inflammatory cluster across tertiles of dairy consumption	The highest tertile of yoghurt consumption was 0.34 [95% CI: (0.14–0.81)] relative to the reference tertile, demonstrating a linear effect (*p*_trend_ = 0.015). Cheese consumption exhibited an OR of 2.49 (95% CI: (1.09–5.75)) relative to the reference	Increasing yoghurt consumption might have a protective effect on inflammation, while cheese consumption seems to be associated with a pro-inflammatory status
van Meijl, 2010 [163]	Netherlands	Randomised crossover	Effects of low-fat milk and yoghurt intake on inflammatory markers in 35 overweight or obese participants versus carbohydrate controls for 8 weeks	No significant effects on IL-6, MCP-1, ICAM-1 or VCAM-1 versus control. TNF-α index decreased by 53 (*p* = 0.015)	Low-fat dairy consumption may increase concentrations of s-TNFR but it has no effects on other inflammatory markers of chronic inflammation and endothelial function
Zemel, 2010 [164]	USA	Randomised crossover	Effects of a dairy-rich, high calcium diet on oxidative and inflammatory stress in 10 overweight and 10 obese individuals compared with soy supplemented eucaloric diets	After 7 days, dairy intake decreased oxidative stress by lowering 8-isoprostane-F_2α_ (12%, *p* < 0.0005), plasma malondialdehyde (22%, *p* < 0.0005). Adiponectin increased significantly (20%, *p* < 0.002). Inflammatory markers were significantly reduced versus the control diet: IL-6 (13%, *p* < 0.01); TNF-α (15%, *p* < 0.002); MCP-1 (10%, *p* < 0.0006)	An increase in dairy food intake produces significant and substantial suppression of the oxidative and inflammatory stress associated with overweight and obesity
Nestel, 2013 [165]	Australia	Randomised crossover	Consumption of full-fat versus low-fat dairy on biomarkers of inflammation in 12 overweigh individuals	75% of those who consumed low-fat products versus full-fat fermented products tended to have higher levels of inflammatory markers tested (CRP, IL-13, TNF-α, VCAM-1 and others; *p*_trend_ < 0.001)	Short-term diets of low-fat dairy products did not lead to a favourable biomarker profile associated with CVD risk compared with the full-fat dairy products. Full-fat fermented dairy products are more favourable
Labonté, 2014 [166]	Canada	Randomised crossover	Assessing the impact of dairy intake versus energy equivalent products on inflammatory markers in 112 healthy participants with systemic inflammation	After dairy consumption, no significant changes in CRP (7.3%, *p* = 0.47). However, both the control and dairy diet reduced IL-6 (17.6% and 19.9%, respectively; *p* < 0.0001 for both, *p* = 0.77 for between-diet comparison	Short-term consumption of a combination of low- and high-fat dairy products as part of a healthy diet has no adverse effects on inflammation
Dugan, 2016 [167]	USA	Randomised crossover	Effect of low-fat dairy consumption on hepatic enzymes and inflammation in 37 participants with metabolic syndrome versus a carbohydrate control	Lower levels of TNF-α (*p* = 0.028) and MCP-1 (*p* = 0.001) were observed in women after low-fat dairy intake versus the control group. The hepatic steatosis index was also reduced (*p* = 0.001)	Three servings of dairy per day improved both liver function and systemic inflammation in subjects with metabolic syndrome
Zemel, 2008 [168]	USA	Randomised controlled longitudinal	Evaluation of feeding calcium rich high-dairy eucaloric diet and hypocaloric diet versus low dairy group intake in obese participants over 24 weeks	High-dairy eucaloric diet and a hypocaloric diet resulted in an 11% (*p* < 0.03) and 29% (*p* < 0.01) decrease in CRP, respectively (post-test vs. pre-test), whereas there was no significant change in the low-dairy groups. Adiponectin decreased by 8% in subjects fed the eucaloric high-dairy diet (*p* = 0.003) and 18% for the hypocaloric high-dairy diet (*p =* 0.05)	Dietary calcium suppresses adipose tissue oxidative and inflammatory stress
de Aguilar-Nascimento, 2011 [169]	Brazil	Randomised controlled longitudinal	Effects of an early enteral formula on the levels of glutathione and inflammatory markers in 25 aged patients with acute ischemic stroke. Group 1 consumed whey, group 2, the control consumed casein	Mortality was similar between groups (33%; *p* = 1.00) and was associated with higher IL-6 levels (group 1: 73.7 ± 24.7; versus group 2: 16.6 ± 2.4 pg/dL; *p =* 0.04) and CRP (82.0 ± 35.6 vs. 48.3 ± 14.5 mg/L; *p* = 0.02). Serum IL-6 was lower (*p* = 0.03) and glutathione was higher (*p* = 0.03) in whey protein-fed patients versus the casein group	Enteral formula containing whey protein may decrease inflammation and increase antioxidant defences in elderly patients with ischemic stroke
Jones, 2013 [170]	Canada	Randomised controlled longitudinal	Assessing a diet rich in calcium and dairy products on weight loss and appetite during energy restriction in 49 overweight and obese individuals for 12 weeks, versus a suitable control. A meal tolerance test was carried out in week 12	MCP-1 was reduced after 30 mins with Dairy/Calcium group compared with the control in the meal tolerance test (*p* = 0.04). No change was observed for IL-6, TNF-α, or IL-1β	Modest reduction in MCP-1
Pei, 2017 [171]	USA	Randomised controlled	Premenopausal women (BMI 18.5–27 and 30–40 kg/m^2^) were randomised to consume 339 g of low-fat yoghurt (yoghurt non-obese (YN); yoghurt obese (YO)) or 324 g of soya pudding (control non-obese; control obese (CO)) daily for 9 weeks (n 30/group). Fasting blood samples were analysed for various inflammatory markers	After 9-week yoghurt consumption, YO and YN had decreased TNF-α/sTNFR RII. Yoghurt consumption increased plasma IgM EndoCAb regardless of obesity status. sCD14 was not affected by diet but LBP/sCD14 was lowered in both YN and YO. Yoghurt intervention increased plasma 2-arachidonoylglycerol in YO but not YN. YO peripheral blood mononuclear cells expression of NF-κB inhibitor α and transforming growth factor β1 increased relative to CO at 9 weeks	Consumption of low-fat yoghurt for 9 weeks reduced biomarkers of chronic inflammation and endotoxin exposure in premenopausal women compared with a non-dairy control food
Wannamethee, 2018 [172]	UK	Prospective cohort study	This study investigated serum CLA (measured as a % of total fatty acids) and the risk of incident heart failure in 3806 older men aged between 60 and 79 years using metabolomics. The men were without prevalent HF and were followed up for an average of 13 years, during which there were 295 incident HF cases	CLA was adversely associated with cholesterol levels but was inversely associated with CRP and NT-proBNP. No association between CLA and CHD. High CLA was associated with reduced risk of HF (hazard ratio [95% confidence interval], 0.64 [0.43–0.96]; quartile 4 versus quartile 1). Elevated CLA was associated with reduced HF risk only in those with higher dairy fat intake, a major dietary source of CLA (*p* = 0.03)	The reduced risk of HF was partially explained by NT-proBNP. High dairy fat intake was not associated with incident coronary heart disease but was associated with reduced risk of HF, largely because of the inverse effect of CLA

BMI = body mass index; CHD = coronary heart disease; CI = confidence interval; CLA = conjugated linoleic acid; CRP = C-reactive protein; EndoCab = endogenous endotoxin-core antibody; HF = heart failure; IL-X = interleukin-β/6/13 etc.; MCP-1 = monocyte chemoattractant protein-1; NF-κB = nuclear factor-κB; NT-proBNP = N-terminal prohormone of brain natriuretic peptide; s-CD14 = soluble cluster of differentiation 14; s-TNFR = soluble tumour necrosis factor receptors; TNF-α = tumour necrosis factor-α; VCAM-1 = vascular cell adhesion molecule-1.

## References

[1] Wilkins E., Wilson L., Wickramasinghe K., Bhatnagar P., Leal J., Luengo-Fernandez R., Burns R., Rayner M., Townsend N. (2017). European Cardiovascular Disease Statistics 2017.

[2] Benjamin E.J., Blaha M.J., Chiuve S.E., Cushman M., Das S.R., Deo R., de Ferranti S.D., Floyd J., Fornage M., Gillespie C. (2017). Heart disease and stroke statistics—2017 update: A report from the american heart association. Circulation.

[3] Health Service Executive Ireland Coronary Heart Disease. http://www.hse.ie/eng/health/az/C/Coronary-heart-disease/.

[4] Lordan R., Zabetakis I. (2017). Invited review: The anti-inflammatory properties of dairy lipids. J. Dairy Sci..

[5] O’Keefe J.H., Gheewala N.M., O’Keefe J.O. (2008). Dietary strategies for improving post-prandial glucose, lipids, inflammation, and cardiovascular health. J. Am. Coll. Cardiol..

[6] Mozaffarian D., Appel L.J., Van Horn L. (2011). Components of a cardioprotective diet new insights. Circulation.

[7] Kapaj A., Deci E., Collier R.J., Preedy V.R. (2017). Chapter 7—World milk production and socio-economic factors effecting its consumption a2—Watson, ronald ross. Dairy in Human Health and Disease Across the Lifespan.

[8] Artaud-Wild S.M., Connor S., Sexton G., Connor W.E. (1993). Differences in coronary mortality can be explained by differences in cholesterol and saturated fat intakes in 40 countries but not in France and Finland. A paradox. Circulation.

[9] Turpeinen O. (1979). Effect of cholesterol-lowering diet on mortality from coronary heart disease and other causes. Circulation.

[10] Huth P.J., Park K.M. (2012). Influence of dairy product and milk fat consumption on cardiovascular disease risk: A review of the evidence. Adv. Nutr..

[11] Grosso G., Collier R.J., Preedy V.R. (2017). Chapter 30—Milk and chronic-degenerative diseases: Main components and potential mechanisms a2—Watson, ronald ross. Dairy in Human Health and Disease Across the Lifespan.

[12] Guo J., Astrup A., Lovegrove J.A., Gijsbers L., Givens D.I., Soedamah-Muthu S.S. (2017). Milk and dairy consumption and risk of cardiovascular diseases and all-cause mortality: Dose-response meta-analysis of prospective cohort studies. Eur. J. Epidemiol..

[13] Goldbohm R.A., Chorus A.M.J., Garre F.G., Schouten L.J., van den Brandt P.A. (2011). Dairy consumption and 10-y total and cardiovascular mortality: A prospective cohort study in The Netherlands. Am. J. Clin. Nutr..

[14] Lamarche B., Givens D.I., Soedamah-Muthu S., Krauss R.M., Jakobsen M.U., Bischoff-Ferrari H.A., Pan A., Després J.-P. (2016). Does milk consumption contribute to cardiometabolic health and overall diet quality?. Can. J. Cardiol..

[15] Dumas A.-A., Lapointe A., Dugrenier M., Provencher V., Lamarche B., Desroches S. (2017). A systematic review of the effect of yogurt consumption on chronic diseases risk markers in adults. Eur. J. Clin. Nutr..

[16] Alexander D.D., Bylsma L.C., Vargas A.J., Cohen S.S., Doucette A., Mohamed M., Irvin S.R., Miller P.E., Watson H., Fryzek J.P. (2016). Dairy consumption and CVD: A systematic review and meta-analysis. Br. J. Nutr..

[17] Qin L.-Q., Xu J.-Y., Han S.-F., Zhang Z.-L., Zhao Y.-Y., Szeto I.M. (2015). Dairy consumption and risk of cardiovascular disease: An updated meta-analysis of prospective cohort studies. Asia Pac. J. Clin. Nutr..

[18] Crichton G.E., Elias M.F. (2014). Dairy food intake and cardiovascular health: The maine-syracuse study. Adv. Dairy Res..

[19] Crichton G.E., Alkerwi A. (2014). Dairy food intake is positively associated with cardiovascular health: Findings from observation of cardiovascular risk factors in Luxembourg study. Nutr. Res..

[20] Gijsbers L., Ding E.L., Malik V.S., de Goede J., Geleijnse J.M., Soedamah-Muthu S.S. (2016). Consumption of dairy foods and diabetes incidence: A dose-response meta-analysis of observational studies. Am. J. Clin. Nutr..

[21] Lu L., Xun P., Wan Y., He K., Cai W. (2016). Long-term association between dairy consumption and risk of childhood obesity: A systematic review and meta-analysis of prospective cohort studies. Eur. J. Clin. Nutr..

[22] Crichton G.E., Alkerwi A. (2014). Whole-fat dairy food intake is inversely associated with obesity prevalence: Findings from the observation of cardiovascular risk factors in Luxembourg study. Nutr. Res..

[23] Lee K., Cho W. (2017). The consumption of dairy products is associated with reduced risks of obesity and metabolic syndrome in Korean women but not in men. Nutrients.

[24] Chen G.C., Szeto I., Chen L., Han S., Li Y., van Hekezen R., Qin L. (2015). Dairy products consumption and metabolic syndrome in adults: Systematic review and meta-analysis of observational studies. Sci. Rep..

[25] Liu A.G., Ford N.A., Hu F.B., Zelman K.M., Mozaffarian D., Kris-Etherton P.M. (2017). A healthy approach to dietary fats: Understanding the science and taking action to reduce consumer confusion. Nutr. J..

[26] Rousseau S. (2015). The celebrity quick-fix. Food Cult. Soc..

[27] United States Department of Health and Human Services (2017). Dietary Guidelines for Americans 2015–2020.

[28] Health Service Executive Ireland Healthy Food for Life Guidelines. http://www.hse.ie/eng/about/Who/healthwellbeing/Our-Priority-Programmes/HEAL/Healthy-Eating-Guidelines/.

[29] Weaver C.M. (2014). How sound is the science behind the dietary recommendations for dairy?. Am. J. Clin. Nutr..

[30] Bantle J.P., Wylie-Rosett J., Albright A.L., Apovian C.M., Clark N.G., Franz M.J., Hoogwerf B.J., Lichtenstein A.H., Mayer-Davis E., Mooradian A.D. (2008). Nutrition recommendations and interventions for diabetes: A position statement of the American Diabetes Association. Diabetes Care.

[31] Benatar J.R., Collier R.J., Preedy V.R. (2017). Chapter 20—Does dairy food have effects on cardiovascular disease and cardiometabolic risk? A2—Watson, ronald ross. Dairy in Human Health and Disease Across the Lifespan.

[32] Harvard School of Public Health Healthy Eating Plate and Healthy Eating Pyramid. https://www.hsph.harvard.edu/nutritionsource/healthy-eating-plate/.

[33] National Health and Medical Research Council, Department of Health and Ageing (2013). Australian Dietary Guidelines Ageing.

[34] Markey O., Vasilopoulou D., Givens D.I., Lovegrove J.A. (2014). Dairy and cardiovascular health: Friend or foe?. Nutr. Bull..

[35] Wade A.T., Davis C.R., Dyer K.A., Hodgson J.M., Woodman R.J., Keage H.A., Murphy K.J. (2017). A mediterranean diet to improve cardiovascular and cognitive health: Protocol for a randomised controlled intervention study. Nutrients.

[36] Van Ballegooijen A.J., Beulens J.W. (2017). The role of vitamin K status in cardiovascular health: Evidence from observational and clinical studies. Curr. Nutr. Rep..

[37] Heaney R.P. (2000). Calcium, dairy products and osteoporosis. J. Am. Coll. Nutr..

[38] Edem D.O. (2002). Palm oil: Biochemical, physiological, nutritional, hematological and toxicological aspects: A review. Plant Foods Hum. Nutr..

[39] Mukherjee S., Mitra A. (2009). Health effects of palm oil. J. Hum. Ecol..

[40] Pehowich D.J., Gomes A.V., Barnes J.A. (2000). Fatty acid composition and possible health effects of coconut constituents. West Indian Med. J..

[41] Visioli F., Strata A. (2014). Milk, dairy products, and their functional effects in humans: A narrative review of recent evidence. Adv. Nutr..

[42] Hooper L., Summerbell C.D., Thompson R., Sills D., Roberts F.G., Moore H., Smith G.D. (2011). Reduced or modified dietary fat for preventing cardiovascular disease. Cochrane Database Syst. Rev..

[43] Micha R., Mozaffarian D. (2010). Saturated fat and cardiometabolic risk factors, coronary heart disease, stroke, and diabetes: A fresh look at the evidence. Lipids.

[44] Jakobsen M.U., O’Reilly E.J., Heitmann B.L., Pereira M.A., Bälter K., Fraser G.E., Goldbourt U., Hallmans G., Knekt P., Liu S. (2009). Major types of dietary fat and risk of coronary heart disease: A pooled analysis of 11 cohort studies. Am. J. Clin. Nutr..

[45] Nettleton J.A., Brouwer I.A., Geleijnse J.M., Hornstra G. (2017). Saturated fat consumption and risk of coronary heart disease and ischemic stroke: A science update. Ann. Nutr. Metab..

[46] Association A.H. (1961). Dietary Fat and Its Relation to Heart Attacks and Strokes.

[47] Kannel W.B., Dawber T.R., Kagan A., Revotskie N., Stokes J. (1961). Factors of risk in the development of coronary heart disease—Six-year follow-up experiencethe Framingham study. Ann. Intern. Med..

[48] La Berge A.F. (2008). How the ideology of low fat conquered America. J. Hist. Med. Allied Sci..

[49] Siri-Tarino P.W., Sun Q., Hu F.B., Krauss R.M. (2010). Meta-analysis of prospective cohort studies evaluating the association of saturated fat with cardiovascular disease. Am. J. Clin. Nutr..

[50] Legrand P., Rioux V. (2010). The complex and important cellular and metabolic functions of saturated fatty acids. Lipids.

[51] Kubow S. (1996). The influence of positional distribution of fatty acids in native, interesterified and structure-specific lipids on lipoprotein metabolism and atherogenesis. J. Nutr. Biochem..

[52] Astrup A., Dyerberg J., Elwood P., Hermansen K., Hu F.B., Jakobsen M.U., Kok F.J., Krauss R.M., Lecerf J.M., LeGrand P. (2011). The role of reducing intakes of saturated fat in the prevention of cardiovascular disease: Where does the evidence stand in 2010?. Am. J. Clin. Nutr..

[53] Lordan R., Tsoupras A., Zabetakis I. (2017). Phospholipids of animal and marine origin: Structure, function, and anti-inflammatory properties. Molecules.

[54] Walz C.P., Barry A.R., Koshman S.L. (2016). Omega-3 polyunsaturated fatty acid supplementation in the prevention of cardiovascular disease. Can. Pharm. J..

[55] Marventano S., Kolacz P., Castellano S., Galvano F., Buscemi S., Mistretta A., Grosso G. (2015). A review of recent evidence in human studies of n-3 and n-6 PUFA intake on cardiovascular disease, cancer, and depressive disorders: Does the ratio really matter?. Int. J. Food Sci. Nutr..

[56] Nettleton J.A., Legrand P., Mensink R.P. (2015). Issfal 2014 debate: It is time to update saturated fat recommendations. Ann. Nutr. Metab..

[57] Livingstone K.M., Lovegrove J.A., Givens D.I. (2012). The impact of substituting SFA in dairy products with MUFA or PUFA on CVD risk: Evidence from human intervention studies. Nutr. Res. Rev..

[58] Markey O., Souroullas K., Fagan C.C., Kliem K.E., Vasilopoulou D., Jackson K.G., Humphries D.J., Grandison A.S., Givens D.I., Lovegrove J.A. (2017). Consumer acceptance of dairy products with a saturated fatty acid–reduced, monounsaturated fatty acid–enriched content. J. Dairy Sci..

[59] Bayat A.R., Tapio I., Vilkki J., Shingfield K.J., Leskinen H. (2018). Plant oil supplements reduce methane emissions and improve milk fatty acid composition in dairy cows fed grass silage-based diets without affecting milk yield. J. Dairy Sci..

[60] Fernandez M.L., West K.L. (2005). Mechanisms by which dietary fatty acids modulate plasma lipids. J. Nutr..

[61] Mensink R.P., Zock P.L., Kester A.D., Katan M.B. (2003). Effects of dietary fatty acids and carbohydrates on the ratio of serum total to HDL cholesterol and on serum lipids and apolipoproteins: A meta-analysis of 60 controlled trials. Am. J. Clin. Nutr..

[62] German J.B., Dillard C.J. (2004). Saturated fats: What dietary intake?. Am. J. Clin. Nutr..

[63] Lawrence G.D. (2013). Dietary fats and health: Dietary recommendations in the context of scientific evidence. Adv. Nutr..

[64] Skeaff C.M., Miller J. (2009). Dietary fat and coronary heart disease: Summary of evidence from prospective cohort and randomised controlled trials. Ann. Nutr. Metab..

[65] Dehghan M., Mente A., Zhang X., Swaminathan S., Li W., Mohan V., Iqbal R., Kumar R., Wentzel-Viljoen E., Rosengren A. (2017). Associations of fats and carbohydrate intake with cardiovascular disease and mortality in 18 countries from five continents (pure): A prospective cohort study. Lancet.

[66] Fernandez M.L., Calle M. (2010). Revisiting dietary cholesterol recommendations: Does the evidence support a limit of 300 mg/d?. Curr. Atheroscler. Rep..

[67] Rong Y., Chen L., Zhu T., Song Y., Yu M., Shan Z., Sands A., Hu F.B., Liu L. (2013). Egg consumption and risk of coronary heart disease and stroke: Dose-response meta-analysis of prospective cohort studies. BMJ.

[68] Krauss R.M., Eckel R.H., Howard B., Appel L.J., Daniels S.R., Deckelbaum R.J., Erdman J.W., Kris-Etherton P., Goldberg I.J., Kotchen T.A. (2000). Aha dietary guidelines. Revision 2000: A Statement for Healthcare professionals From the Nutrition Committee of the American Heart Association. Circulation.

[69] Genest J., McPherson R., Frohlich J., Anderson T., Campbell N., Carpentier A., Couture P., Dufour R., Fodor G., Francis G.A. (2009). 2009 canadian cardiovascular society/canadian guidelines for the diagnosis and treatment of dyslipidemia and prevention of cardiovascular disease in the adult—2009 recommendations. Can. J. Cardiol..

[70] Public Health England (2016). The Eatwell Guide—Government Dietary Recommendations: Government Recommendations for Energy and Nutrients for Males and Females Aged 1–18 Years and 19+ Years.

[71] Jang Y.-A., Lee H.-S., Kim B.-H., Lee Y.-N., Lee H.-J., Moon J.-J., Kim C.-I. (2008). Revised dietary guidelines for Koreans. Asia Pac. J. Clin. Nutr..

[72] Ministry of Health (2003). Food and Nutrition Guidelines for Healthy Adults: A Background Paper.

[73] McNamara D.J. (1997). Cholesterol intake and plasma cholesterol: An update. J. Am. Coll. Nutr..

[74] Hu F.B., Stampfer M.J., Rimm E.B., Manson J.E., Ascherio A., Colditz G.A., Rosner B.A., Spiegelman D., Speizer F.E., Sacks F.M. (1999). A prospective study of egg consumption and risk of cardiovascular disease in men and women. JAMA.

[75] Esrey K.L., Joseph L., Grover S.A. (1996). Relationship between dietary intake and coronary heart disease mortality: Lipid research clinics prevalence follow-up study. J. Clin. Epidemiol..

[76] Herron K.L., Vega-Lopez S., Conde K., Ramjiganesh T., Roy S., Shachter N.S., Fernandez M.L. (2002). Pre-menopausal women, classified as hypo- or hyper-responders, do not alter their LDL/HDL ratio following a high dietary cholesterol challenge. J. Am. Coll. Nutr..

[77] Fernandez M.L., Webb D. (2008). The LDL to HDL cholesterol ratio as a valuable tool to evaluate coronary heart disease risk. J. Am. Coll. Nutr..

[78] Blesso C. (2015). Egg phospholipids and cardiovascular health. Nutrients.

[79] Mann G.V., Spoerry A. (1974). Studies of a surfactant and cholesteremia in the Maasai. Am. J. Clin. Nutr..

[80] Steinmetz K.A., Childs M.T., Stimson C., Kushi L.H., McGovern P.G., Potter J.D., Yamanaka W.K. (1994). Effect of consumption of whole milk and skim milk on blood lipid profiles in healthy men. Am. J. Clin. Nutr..

[81] Sharpe S.J., Gamble G.D., Sharpe D.N. (1994). Cholesterol-lowering and blood pressure effects of immune milk. Am. J. Clin. Nutr..

[82] Buonopane G.J., Kilara A., Smith J.S., McCarthy R.D. (1992). Effect of skim milk supplementation on blood cholesterol concentration, blood pressure, and triglycerides in a free-living human population. J. Am. Coll. Nutr..

[83] St-Onge M.-P., Farnworth E.R., Jones P.J.H. (2000). Consumption of fermented and nonfermented dairy products: Effects on cholesterol concentrations and metabolism. Am. J. Clin. Nutr..

[84] Tholstrup T., Høy C.-E., Andersen L.N., Christensen R.D.K., Sandström B. (2004). Does fat in milk, butter and cheese affect blood lipids and cholesterol differently?. J. Am. Coll. Nutr..

[85] Biong A.S., Muller H., Seljeflot I., Veierod M.B., Pedersen J.I. (2004). A comparison of the effects of cheese and butter on serum lipids, haemostatic variables and homocysteine. Br. J. Nutr..

[86] Nestel P.J., Chronopulos A., Cehun M. (2005). Dairy fat in cheese raises LDL cholesterol less than that in butter in mildly hypercholesterolaemic subjects. Eur. J. Clin. Nutr..

[87] Hjerpsted J., Leedo E., Tholstrup T. (2011). Cheese intake in large amounts lowers LDL-cholesterol concentrations compared with butter intake of equal fat content. Am. J. Clin. Nutr..

[88] Brassard D., Tessier-Grenier M., Allaire J., Rajendiran E., She Y., Ramprasath V., Gigleux I., Talbot D., Levy E., Tremblay A. (2017). Comparison of the impact of SFAs from cheese and butter on cardiometabolic risk factors: A randomized controlled trial. Am. J. Clin. Nutr..

[89] Lorenzen J.K., Astrup A. (2011). Dairy calcium intake modifies responsiveness of fat metabolism and blood lipids to a high-fat diet. Br. J. Nutr..

[90] Givens D.I. (2017). Saturated fats, dairy foods and health: A curious paradox?. Nutr. Bull..

[91] Hjerpsted J.B., Dragsted L.O., Tholstrup T. (2016). Cheese intake lowers plasma cholesterol concentrations without increasing bile acid excretion. J. Nutr. Intermed. Metab..

[92] Walsh B. (2014). The truth about fat. Time Magazine.

[93] Matthan N.R., Welty F.K., Barrett P.H.R., Harausz C., Dolnikowski G.G., Parks J.S., Eckel R.H., Schaefer E.J., Lichtenstein A.H. (2004). Dietary hydrogenated fat increases high-density lipoprotein apoA-I catabolism and decreases low-density lipoprotein apoB-100 catabolism in hypercholesterolemic women. Arterioscler. Thromb. Vasc. Biol..

[94] Zock P.L., Katan M.B. (1997). Butter, margarine and serum lipoproteins. Atherosclerosis.

[95] Pimpin L., Wu J.H., Haskelberg H., Del Gobbo L., Mozaffarian D. (2016). Is butter back? A systematic review and meta-analysis of butter consumption and risk of cardiovascular disease, diabetes, and total mortality. PLoS ONE.

[96] United States Senate Select Committee on Nutrition and Human Needs (1977). Dietary Goals for the United States, Supplemental Views.

[97] Reedy J. (2016). How the US Low-Fat Diet Recommendations of 1977 Contributed to the Declining Health of Americans. Ph.D. Thesis.

[98] Ludwig D.S. (2016). Lowering the bar on the low-fat diet. JAMA.

[99] Wang H., Fox C.S., Troy L.M., Mckeown N.M., Jacques P.F. (2015). Longitudinal association of dairy consumption with the changes in blood pressure and the risk of incident hypertension: The framingham heart study. Br. J. Nutr..

[100] Ralston R.A., Lee J.H., Truby H., Palermo C.E., Walker K.Z. (2012). A systematic review and meta-analysis of elevated blood pressure and consumption of dairy foods. J. Hum. Hypertens..

[101] Soedamah-Muthu S.S., Verberne L.D.M., Ding E.L., Engberink M.F., Geleijnse J.M. (2012). Dairy consumption and incidence of hypertension: A dose-response meta-analysis of prospective cohort studies. Hypertension.

[102] Drouin-Chartier J.-P., Brassard D., Tessier-Grenier M., Côté J.A., Labonté M.-È., Desroches S., Couture P., Lamarche B. (2016). Systematic review of the association between dairy product consumption and risk of cardiovascular-related clinical outcomes. Adv. Nutr..

[103] Appel L.J., Brands M.W., Daniels S.R., Karanja N., Elmer P.J., Sacks F.M. (2006). Dietary approaches to prevent and treat hypertension: A Scientific Statement From the American Heart Association. Hypertension.

[104] Da Silva M.S., Rudkowska I. (2014). Dairy products on metabolic health: Current research and clinical implications. Maturitas.

[105] Alonso A., Zozaya C., Vázquez Z., Alfredo Martínez J., Martínez-González M.A. (2009). The effect of low-fat versus whole-fat dairy product intake on blood pressure and weight in young normotensive adults. J. Hum. Nutr. Diet..

[106] Spence L.A., Cifelli C.J., Miller G.D. (2011). The role of dairy products in healthy weight and body composition in children and adolescents. Curr. Nutr. Food Sci..

[107] Vanderhout S.M., Birken C.S., Parkin P.C., Lebovic G., Chen Y., O’Connor D.L., Maguire J.L., Collaboration T.T.K. (2016). Relation between milk-fat percentage, vitamin D, and BMI *z* score in early childhood. Am. J. Clin. Nutr..

[108] García Yu I.A.-L., Sánchez-Aguadero N., Recio-Rodríguez J.I., Collier R.J., Preedy V.R. (2017). Chapter 25—Effect of the fat component of dairy products in cardiovascular health, vascular structure and function a2—Watson, ronald ross. Nutrients in Dairy and Their Implications on Health and Disease.

[109] Drehmer M., Pereira M.A., Schmidt M.I., Alvim S., Lotufo P.A., Luft V.C., Duncan B.B. (2015). Total and full-fat, but not low-fat, dairy product intakes are inversely associated with metabolic syndrome in adults. J. Nutr..

[110] Eussen S.J.P.M., van Dongen M.C.J.M., Wijckmans N., den Biggelaar L., Oude Elferink S.J.W.H., Singh-Povel C.M., Schram M.T., Sep S.J.S., van der Kallen C.J., Koster A. (2016). Consumption of dairy foods in relation to impaired glucose metabolism and type 2 diabetes mellitus: The Maastricht study. Br. J. Nutr..

[111] Soedamah-Muthu S.S., Ding E.L., Al-Delaimy W.K., Hu F.B., Engberink M.F., Willett W.C., Geleijnse J.M. (2011). Milk and dairy consumption and incidence of cardiovascular diseases and all-cause mortality: Dose-response meta-analysis of prospective cohort studies. Am. J. Clin. Nutr..

[112] Tremblay A., Gilbert J.-A. (2009). Milk products, insulin resistance syndrome and type 2 diabetes. J. Am. Coll. Nutr..

[113] Thorning T.K., Bertram H.C., Bonjour J.-P., De Groot L., Dupont D., Feeney E., Ipsen R., Lecerf J.M., Mackie A., McKinley M.C. (2017). Whole dairy matrix or single nutrients in assessment of health effects: Current evidence and knowledge gaps. Am. J. Clin. Nutr..

[114] Guarner V., Rubio-Ruiz M.E. (2014). Low-grade systemic inflammation connects aging, metabolic syndrome and cardiovascular disease. Aging and Health—A Systems Biology Perspective.

[115] Vishnu A., Gurka M.J., DeBoer M.D. (2015). The severity of the metabolic syndrome increases over time within individuals, independent of baseline metabolic syndrome status and medication use: The atherosclerosis risk in communities study. Atherosclerosis.

[116] Givens D.I., Livingstone K.M., Pickering J.E., Fekete Á.A., Dougkas A., Elwood P.C. (2014). Milk: White elixir or white poison? An examination of the associations between dairy consumption and disease in human subjects. Anim. Front..

[117] Laurent S., Boutouyrie P., Asmar R., Gautier I., Laloux B., Guize L., Ducimetiere P., Benetos A. (2001). Aortic stiffness is an independent predictor of all-cause and cardiovascular mortality in hypertensive patients. Hypertension.

[118] Livingstone K.M., Lovegrove J.A., Cockcroft J.R., Elwood P.C., Pickering J.E., Givens D.I. (2013). Does dairy food intake predict arterial stiffness and blood pressure in men? Evidence from the caerphilly prospective study. Hypertension.

[119] Appel L.J., Moore T.J., Obarzanek E., Vollmer W.M., Svetkey L.P., Sacks F.M., Bray G.A., Vogt T.M., Cutler J.A., Windhauser M.M. (1997). A clinical trial of the effects of dietary patterns on blood pressure. Dash collaborative research group. N. Engl. J. Med..

[120] Van Meijl L.E., Mensink R.P. (2011). Low-fat dairy consumption reduces systolic blood pressure, but does not improve other metabolic risk parameters in overweight and obese subjects. Nutr. Metab. Cardiovasc. Dis..

[121] Buendia J.R., Li Y., Hu F.B., Cabral H.J., Bradlee M.L., Quatromoni P.A., Singer M.R., Curhan G.C., Moore L.L. (2018). Regular yogurt intake and risk of cardiovascular disease among hypertensive adults. Am. J. Hypertens..

[122] Gholami F., Khoramdad M., Esmailnasab N., Moradi G., Nouri B., Safiri S., Alimohamadi Y. (2017). The effect of dairy consumption on the prevention of cardiovascular diseases: A meta-analysis of prospective studies. J. Cardiovasc. Thorac. Res..

[123] Beltrán-Barrientos L.M., Hernández-Mendoza A., Torres-Llanez M.J., González-Córdova A.F., Vallejo-Córdoba B. (2016). Invited review: Fermented milk as antihypertensive functional food. J. Dairy Sci..

[124] O’Keeffe M.B., FitzGerald R.J. (2018). Whey protein hydrolysate induced modulation of endothelial cell gene expression. J. Funct. Foods.

[125] Morio B., Fardet A., Legrand P., Lecerf J.-M. (2016). Involvement of dietary saturated fats, from all sources or of dairy origin only, in insulin resistance and type 2 diabetes. Nutr. Rev..

[126] Elwood P.C., Pickering J.E., Fehily A.M. (2007). Milk and dairy consumption, diabetes and the metabolic syndrome: The caerphilly prospective study. J. Epidemiol. Community Health.

[127] Choi H.K., Willett W.C., Stampfer M.J., Rimm E., Hu F.B. (2005). Dairy consumption and risk of type 2 diabetes mellitus in men: A prospective study. Arch. Intern. Med..

[128] Crichton G.E., Bryan J., Buckley J., Murphy K.J. (2011). Dairy consumption and metabolic syndrome: A systematic review of findings and methodological issues. Obes. Rev..

[129] Fumeron F., Lamri A., Abi Khalil C., Jaziri R., Porchay-Baldérelli I., Lantieri O., Vol S., Balkau B., Marre M., The Data from the Epidemiological Study on the Insulin Resistance Syndrome Study Group (2011). Dairy consumption and the incidence of hyperglycemia and the metabolic syndrome: Results from a french prospective study, data from the epidemiological study on the insulin resistance syndrome (DESIR). Diabetes Care.

[130] Benatar J.R., Sidhu K., Stewart R.A.H. (2013). Effects of high and low fat dairy food on cardio-metabolic risk factors: A meta-analysis of randomized studies. PLoS ONE.

[131] Tong X., Dong J.Y., Wu Z.W., Li W., Qin L.Q. (2011). Dairy consumption and risk of type 2 diabetes mellitus: A meta-analysis of cohort studies. Eur. J. Clin. Nutr..

[132] Chen M., Sun Q., Giovannucci E., Mozaffarian D., Manson J.E., Willett W.C., Hu F.B. (2014). Dairy consumption and risk of type 2 diabetes: 3 cohorts of us adults and an updated meta-analysis. BMC Med..

[133] Gao D., Ning N., Wang C., Wang Y., Li Q., Meng Z., Liu Y., Li Q. (2013). Dairy products consumption and risk of type 2 diabetes: Systematic review and dose-response meta-analysis. PLoS ONE.

[134] Aune D., Norat T., Romundstad P., Vatten L.J. (2013). Dairy products and the risk of type 2 diabetes: A systematic review and dose-response meta-analysis of cohort studies. Am. J. Clin. Nutr..

[135] Bergholdt H.K.M., Nordestgaard B.G., Ellervik C. (2015). Milk intake is not associated with low risk of diabetes or overweight-obesity: A mendelian randomization study in 97,811 Danish individuals. Am. J. Clin. Nutr..

[136] Sluijs I., Forouhi N.G., Beulens J.W.J., van der Schouw Y.T., Agnoli C., Arriola L., Balkau B., Barricarte A., Boeing H., Bueno-de-Mesquita H.B. (2012). The amount and type of dairy product intake and incident type 2 diabetes: Results from the epic-interact study. Am. J. Clin. Nutr..

[137] Clifton P., Collier R.J., Preedy V.R. (2017). Chapter 32—The influence of dairy consumption on the risk of type 2 diabetes, metabolic syndrome, and impaired glucose tolerance or insulin resistance: A review of cohort and intervention studies a2—Watson, ronald ross. Dairy in Human Health and Disease Across the Lifespan.

[138] Parodi P.W. (2016). Cooperative action of bioactive components in milk fat with ppars may explain its anti-diabetogenic properties. Med. Hypotheses.

[139] Keast D., Hill Gallant K., Albertson A., Gugger C., Holschuh N. (2015). Associations between yogurt, dairy, calcium, and vitamin D intake and obesity among U.S. Children aged 8–18 years: NHANES, 2005–2008. Nutrients.

[140] Walsh B., Cullinan J. (2015). Decomposing socioeconomic inequalities in childhood obesity: Evidence from Ireland. Econ. Hum. Biol..

[141] Moore L.L., Singer M.R., Qureshi M.M., Bradlee M.L. (2008). Dairy intake and anthropometric measures of body fat among children and adolescents in NHANES. J. Am. Coll. Nutr..

[142] Bradlee M.L., Singer M.R., Qureshi M.M., Moore L.L. (2009). Food group intake and central obesity among children and adolescents in the third national health and nutrition examination survey (NHANES III). Public Health Nutr..

[143] Wiley A.S. (2010). Dairy and milk consumption and child growth: Is BMI involved? An analysis of NHANES 1999–2004. Am. J. Hum. Biol..

[144] Sayon-Orea C., Martínez-González M.A., Ruiz-Canela M., Bes-Rastrollo M. (2017). Associations between yogurt consumption and weight gain and risk of obesity and metabolic syndrome: A systematic review. Adv. Nutr..

[145] Da Silva M.S., Rudkowska I. (2015). Dairy nutrients and their effect on inflammatory profile in molecular studies. Mol. Nutr. Food Res..

[146] Palur Ramakrishnan A.V.K., Varghese T.P., Vanapalli S., Nair N.K., Mingate M.D. (2017). Platelet activating factor: A potential biomarker in acute coronary syndrome?. Cardiovasc. Ther..

[147] Castro Faria Neto H.C., Stafforini D.M., Prescott S.M., Zimmerman G.A. (2005). Regulating inflammation through the anti-inflammatory enzyme platelet-activating factor-acetylhydrolase. Mem. Inst. Oswaldo Cruz.

[148] Lordan R., Zabetakis I. (2017). Ovine and caprine lipids promoting cardiovascular health in milk and its derivatives. Adv. Dairy Res.

[149] Antonopoulou S., Semidalas C.E., Koussissis S., Demopoulos C.A. (1996). Platelet-activating factor (PAF) antagonists in foods: A study of lipids with PAF or anti-PAF-like activity in cow’s milk and yogurt. J. Agric. Food Chem..

[150] Poutzalis S., Anastasiadou A., Nasopoulou C., Megalemou K., Sioriki E., Zabetakis I. (2016). Evaluation of the in vitro anti-atherogenic activities of goat milk and goat dairy products. Dairy Sci. Technol..

[151] Tsorotioti S.E., Nasopoulou C., Detopoulou M., Sioriki E., Demopoulos C.A., Zabetakis I. (2014). In vitro anti-atherogenic properties of traditional Greek cheese lipid fractions. Dairy Sci. Technol..

[152] Megalemou K., Sioriki E., Lordan R., Dermiki M., Nasopoulou C., Zabetakis I. (2017). Evaluation of sensory and in vitro anti-thrombotic properties of traditional Greek yogurts derived from different types of milk. Heliyon.

[153] Da Silva M.S., Rudkowska I., Collier R.J., Preedy V.R. (2017). Chapter 22—Macro components in dairy and their effects on inflammation parameters: Preclinical studies a2—Watson, ronald ross. Nutrients in Dairy and Their Implications on Health and Disease.

[154] Stancliffe R.A., Thorpe T., Zemel M.B. (2011). Dairy attentuates oxidative and inflammatory stress in metabolic syndrome. Am. J. Clin. Nutr..

[155] Thompson W.G., Holdman N.R., Janzow D.J., Slezak J.M., Morris K.L., Zemel M.B. (2005). Effect of energy-reduced diets high in dairy products and fiber on weight loss in obese adults. Obes. Res..

[156] Sofi F., Buccioni A., Cesari F., Gori A.M., Minieri S., Mannini L., Casini A., Gensini G.F., Abbate R., Antongiovanni M. (2010). Effects of a dairy product (pecorino cheese) naturally rich in *cis*-9, *trans*-11 conjugated linoleic acid on lipid, inflammatory and haemorheological variables: A dietary intervention study. Nutr. Metab. Cardiovasc. Dis..

[157] Rosado J.L., Garcia O.P., Ronquillo D., Hervert-Hernández D., Caamaño M.D.C., Martínez G., Gutiérrez J., García S. (2011). Intake of milk with added micronutrients increases the effectiveness of an energy-restricted diet to reduce body weight: A randomized controlled clinical trial in Mexican women. J. Am. Diet. Assoc..

[158] Nestel P.J., Pally S., MacIntosh G.L., Greeve M.A., Middleton S., Jowett J., Meikle P.J. (2012). Circulating inflammatory and atherogenic biomarkers are not increased following single meals of dairy foods. Eur. J. Clin. Nutr..

[159] Esmaillzadeh A., Azadbakht L. (2010). Dairy consumption and circulating levels of inflammatory markers among Iranian women. Public Health Nutr..

[160] Panagiotakos D.B., Pitsavos C.H., Zampelas A.D., Chrysohoou C.A., Stefanadis C.I. (2010). Dairy products consumption is associated with decreased levels of inflammatory markers related to cardiovascular disease in apparently healthy adults: The ATTICA study. J. Am. Coll. Nutr..

[161] Wang H., Steffen L.M., Vessby B., Basu S., Steinberger J., Moran A., Jacobs D.R., Hong C.-P., Sinaiko A.R. (2011). Obesity modifies the relations between serum markers of dairy fats and inflammation and oxidative stress among adolescents. Obesity.

[162] Gadotti T.N., Norde M.M., Rogero M.M., Fisberg M., Fisberg R.M., Oki E., Martini L.A. (2017). Dairy consumption and inflammatory profile: A cross-sectional population-based study, São Paulo, Brazil. Nutrition.

[163] Van Meijl L.E.C., Mensink R.P. (2010). Effects of low-fat dairy consumption on markers of low-grade systemic inflammation and endothelial function in overweight and obese subjects: An intervention study. Br. J. Nutr..

[164] Zemel M.B., Sun X., Sobhani T., Wilson B. (2010). Effects of dairy compared with soy on oxidative and inflammatory stress in overweight and obese subjects. Am. J. Clin. Nutr..

[165] Nestel P.J., Mellett N., Pally S., Wong G., Barlow C.K., Croft K., Mori T.A., Meikle P.J. (2013). Effects of low-fat or full-fat fermented and non-fermented dairy foods on selected cardiovascular biomarkers in overweight adults. Br. J. Nutr..

[166] Labonté M.-È., Cyr A., Abdullah M.M., Lépine M.-C., Vohl M.-C., Jones P., Couture P., Lamarche B. (2014). Dairy product consumption has no impact on biomarkers of inflammation among men and women with low-grade systemic inflammation. J. Nutr..

[167] Dugan C.E., Aguilar D., Park Y.K., Lee J.Y., Fernandez M.L. (2016). Dairy consumption lowers systemic inflammation and liver enzymes in typically low-dairy consumers with clinical characteristics of metabolic syndrome. J. Am. Coll. Nutr..

[168] Zemel M.B., Sun X. (2008). Dietary calcium and dairy products modulate oxidative and inflammatory stress in mice and humans. J. Nutr..

[169] De Aguilar-Nascimento J.E., Prado Silveira B.R., Dock-Nascimento D.B. (2011). Early enteral nutrition with whey protein or casein in elderly patients with acute ischemic stroke: A double-blind randomized trial. Nutrition.

[170] Jones K.W., Eller L.K., Parnell J.A., Doyle-Baker P.K., Edwards A.L., Reimer R.A. (2013). Effect of a dairy-and calcium-rich diet on weight loss and appetite during energy restriction in overweight and obese adults: A randomized trial. Eur. J. Clin. Nutr..

[171] Pei R., DiMarco D.M., Putt K.K., Martin D.A., Gu Q., Chitchumroonchokchai C., White H.M., Scarlett C.O., Bruno R.S., Bolling B.W. (2017). Low-fat yogurt consumption reduces biomarkers of chronic inflammation and inhibits markers of endotoxin exposure in healthy premenopausal women: A randomised controlled trial. Br. J. Nutr..

[172] Wannamethee S.G., Jefferis B.J., Lennon L., Papacosta O., Whincup P.H., Hingorani A.D. (2018). Serum conjugated linoleic acid and risk of incident heart failure in older men: The British regional heart study. JAMA.

[173] De Souza R.J., Mente A., Maroleanu A., Cozma A.I., Ha V., Kishibe T., Uleryk E., Budylowski P., Schünemann H., Beyene J. (2015). Intake of saturated and *trans* unsaturated fatty acids and risk of all cause mortality, cardiovascular disease, and type 2 diabetes: Systematic review and meta-analysis of observational studies. BMJ.

[174] Poudyal H., Brown L. (2015). Should the pharmacological actions of dietary fatty acids in cardiometabolic disorders be classified based on biological or chemical function?. Prog. Lipid Res..

[175] Micha R., Mozaffarian D. (2008). *Trans* fatty acids: Effects on cardiometabolic health and implications for policy. Prostaglandins Leukot. Essent. Fat. Acids.

[176] Ganguly R., Pierce G.N. (2012). *Trans* fat involvement in cardiovascular disease. Mol. Nutr. Food Res..

[177] Stone N.J., Robinson J., Lichtenstein A.H., Merz C.N.B., Blum C.B., Eckel R.H., Goldberg A.C., Gordon D., Levy D., Lloyd-Jones D.M. (2013). 2013 ACC/AHA Guideline on the Treatment of Blood Cholesterol to Reduce Atherosclerotic Cardiovascular Risk in Adults.

[178] Lichtenstein A.H., Appel L.J., Brands M., Carnethon M., Daniels S., Franch H.A., Franklin B., Kris-Etherton P., Harris W.S., Howard B. (2006). Diet and lifestyle recommendations revision 2006. Circulation.

[179] L’Abbé M.R., Stender S., Skeaff C.M., Ghafoorunissa, Tavella M. (2009). Approaches to removing *trans* fats from the food supply in industrialized and developing countries. Eur. J. Clin. Nutr..

[180] Dawczynski C., Lorkowski S. (2016). *Trans*-fatty acids and cardiovascular risk: Does origin matter?. Expert Rev. Cardiovasc. Ther..

[181] Mozaffarian D., Katan M.B., Ascherio A., Stampfer M.J., Willett W.C. (2006). *Trans* fatty acids and cardiovascular disease. N. Engl. J. Med..

[182] Wang Y., Jacome-Sosa M.M., Proctor S.D. (2012). The role of ruminant *trans* fat as a potential nutraceutical in the prevention of cardiovascular disease. Food Res. Int..

[183] Lichtenstein A.H. (2014). Dietary *trans* fatty acids and cardiovascular disease risk: Past and present. Curr. Atheroscler. Rep..

[184] Ascherio A., Hennekens C.H., Buring J.E., Master C., Stampfer M.J., Willett W.C. (1994). *Trans*-fatty acids intake and risk of myocardial infarction. Circulation.

[185] Gebauer S.K., Chardigny J.-M., Jakobsen M.U., Lamarche B., Lock A.L., Proctor S.D., Baer D.J. (2011). Effects of ruminant *trans* fatty acids on cardiovascular disease and cancer: A comprehensive review of epidemiological, clinical, and mechanistic studies. Adv. Nutr..

[186] Bassett C.M.C., Edel A.L., Patenaude A.F., McCullough R.S., Blackwood D.P., Chouinard P.Y., Paquin P., Lamarche B.T., Pierce G.N. (2010). Dietary vaccenic acid has antiatherogenic effects in LDLr^−/−^ mice. J. Nutr..

[187] Van de Vijver L., Kardinaal A., Couet C., Aro A. (2000). Association between *trans* fatty acid intake and cardiovascular risk factors in Europe: The transfair study. Eur. J. Clin. Nutr..

[188] Martínez-González M.A., Salas-Salvadó J., Estruch R., Corella D., Fitó M., Ros E. (2015). Benefits of the mediterranean diet: Insights from the predimed study. Prog. Cardiovasc. Dis..

[189] Smit L.A., Baylin A., Campos H. (2010). Conjugated linoleic acid in adipose tissue and risk of myocardial infarction. Am. J. Clin. Nutr..

[190] Moloney F., Toomey S., Noone E., Nugent A., Allan B., Loscher C.E., Roche H.M. (2007). Antidiabetic effects of *cis*-9, *trans*-11–conjugated linoleic acid may be mediated via anti-inflammatory effects in white adipose tissue. Diabetes.

[191] Ahn I.-S., Choi B.-H., Ha J.-H., Byun J.-M., Shin H.-G., Park K.-Y., Do M.-S. (2006). Isomer-specific effect of conjugated linoleic acid on inflammatory adipokines associated with fat accumulation in 3T3-L1 adipocytes. J. Med. Food.

[192] Mozaffarian D., Cao H., King I.B., Lemaitre R.N., Song X., Siscovick D.S., Hotamisligil G.K.S. (2010). *Trans*-palmitoleic acid, metabolic risk factors, and new-onset diabetes in us adults. Ann. Intern. Med..

[193] Bolton-Smith C., Woodward M., Fenton S., Brown C. (1996). Does dietary *trans* fatty acid intake relate to the prevalence of coronary heart disease in Scotland?. Eur. Heart J..

[194] Olmedilla-Alonso B., Nova-Rebato E., García-González N., Martín-Diana A.-B., Fontecha J., Delgado D., Gredilla A.-E., Bueno F., Asensio-Vegas C. (2017). Effect of ewe’s (semi-skimmed and whole) and cow’s milk yogurt consumption on the lipid profile of control subjects: A crossover study. Food Nutr. Res..

[195] Zoumpopoulou G., Pot B., Tsakalidou E., Papadimitriou K. (2017). Dairy probiotics: Beyond the role of promoting gut and immune health. Int. Dairy J..

[196] Elwood P.C., Pickering J.E., Givens D.I., Gallacher J.E. (2010). The consumption of milk and dairy foods and the incidence of vascular disease and diabetes: An overview of the evidence. Lipids.

[197] Darmon N., Drewnowski A. (2008). Does social class predict diet quality?. Am. J. Clin. Nutr..

[198] Hobbs D.A., Givens D.I., Lovegrove J.A. (2018). Yogurt consumption is associated with higher nutrient intake, diet quality and favourable metabolic profle in children: A cross-sectional analysis using data from years 1–4 of the national diet and nutrition survey, UK. Eur. J. Nutr..

[199] Tognon G., Nilsson L.M., Shungin D., Lissner L., Jansson J.-H., Renström F., Wennberg M., Winkvist A., Johansson I. (2017). Nonfermented milk and other dairy products: Associations with all-cause mortality. Am. J. Clin. Nutr..

[200] Tong X., Chen G.-C., Zhang Z., Wei Y.-L., Xu J.-Y., Qin L.-Q. (2017). Cheese consumption and risk of all-cause mortality: A meta-analysis of prospective studies. Nutrients.

[201] Kießling G., Schneider J., Jahreis G. (2002). Long-term consumption of fermented dairy products over 6 months increases HDL cholesterol. Eur. J. Clin. Nutr..

[202] Turner K.M., Keogh J.B., Clifton P.M. (2015). Dairy consumption and insulin sensitivity: A systematic review of short- and long-term intervention studies. Nutr. Metab. Cardiovasc. Dis..

[203] Hove K.D., Brøns C., Færch K., Lund S.S., Rossing P., Vaag A. (2015). Effects of 12 weeks of treatment with fermented milk on blood pressure, glucose metabolism and markers of cardiovascular risk in patients with type 2 diabetes: A randomised double-blind placebo-controlled study. Eur. J. Endocrinol..

[204] Houston D.K., Driver K.E., Bush A.J., Kritchevsky S.B. (2008). The association between cheese consumption and cardiovascular risk factors among adults. J. Hum. Nutr. Diet..

[205] Tholstrup T. (2006). Dairy products and cardiovascular disease. Curr. Opin. Lipidol..

[206] Tavani A., Gallus S., Negri E., La Vecchia C. (2002). Milk, dairy products, and coronary heart disease. J. Epidemiol. Community Health.

[207] Hjerpsted J., Tholstrup T. (2016). Cheese and cardiovascular disease risk: A review of the evidence and discussion of possible mechanisms. Crit. Rev. Food Sci. Nutr..

[208] Praagman J., Dalmeijer G.W., van der Schouw Y.T., Soedamah-Muthu S.S., Monique Verschuren W.M., Bas Bueno-de-Mesquita H., Geleijnse J.M., Beulens J.W.J. (2015). The relationship between fermented food intake and mortality risk in the European prospective investigation into cancer and nutrition-netherlands cohort. Br. J. Nutr..

[209] Wu L., Sun D. (2017). Consumption of yogurt and the incident risk of cardiovascular disease: A meta-analysis of nine cohort studies. Nutrients.

[210] Sayón-Orea C., Bes-Rastrollo M., Martí A., Pimenta A.M., Martín-Calvo N., Martínez-González M.A. (2015). Association between yogurt consumption and the risk of metabolic syndrome over 6 years in the sun study. BMC Public Health.

[211] Renaud S., de Lorgeril M. (1992). Wine, alcohol, platelets, and the french paradox for coronary heart disease. Lancet.

[212] Lallès J.-P. (2016). Dairy products and the french paradox: Could alkaline phosphatases play a role?. Med. Hypotheses.

[213] Petyaev I.M., Bashmakov Y.K. (2012). Could cheese be the missing piece in the French paradox puzzle?. Med. Hypotheses.

[214] Parvez S., Malik K.A., Ah Kang S., Kim H.Y. (2006). Probiotics and their fermented food products are beneficial for health. J. Appl. Microbiol..

[215] Booth S.L. (2009). Roles for vitamin K beyond coagulation. Annu. Rev. Nutr..

[216] O’Connor E.M., Durack E. (2017). Osteocalcin: The extra-skeletal role of a vitamin K-dependent protein in glucose metabolism. J. Nutr. Intermed. Metab..

[217] Walther B., Karl J.P., Booth S.L., Boyaval P. (2013). Menaquinones, bacteria, and the food supply: The relevance of dairy and fermented food products to vitamin K requirements. Adv. Nutr..

[218] Booth S.L., Rajabi A.A. (2008). Determinants of vitamin K status in humans. Vitam. Horm..

[219] Fu X., Harshman S.G., Shen X., Haytowitz D.B., Karl J.P., Wolfe B.E., Booth S.L. (2017). Multiple vitamin K forms exist in dairy foods. Curr. Dev. Nutr..

[220] Shea M.K., Holden R.M. (2012). Vitamin K status and vascular calcification: Evidence from observational and clinical studies. Adv. Nutr..

[221] Harshman S.G., Shea M.K. (2016). The role of vitamin K in chronic aging diseases: Inflammation, cardiovascular disease, and osteoarthritis. Curr. Nutr. Rep..

[222] Gast G.C.M., de Roos N.M., Sluijs I., Bots M.L., Beulens J.W.J., Geleijnse J.M., Witteman J.C., Grobbee D.E., Peeters P.H.M., van der Schouw Y.T. (2009). A high menaquinone intake reduces the incidence of coronary heart disease. Nutr. Metab. Cardiovasc. Dis..

[223] Nagata C., Wada K., Tamura T., Konishi K., Goto Y., Koda S., Kawachi T., Tsuji M., Nakamura K. (2017). Dietary soy and natto intake and cardiovascular disease mortality in Japanese adults: The Takayama study. Am. J. Clin. Nutr..

[224] Keyzer C.A., Vermeer C., Joosten M.M., Knapen M.H., Drummen N.E., Navis G., Bakker S.J., de Borst M.H. (2015). Vitamin K status and mortality after kidney transplantation: A cohort study. Am. J. Kidney Dis..

[225] Rosa D.D., Dias M.M.S., Grześkowiak Ł.M., Reis S.A., Conceição L.L., Peluzio M.D.C.G. (2017). Milk *kefir*: Nutritional, microbiological and health benefits. Nutr. Res. Rev..

[226] Nestel P., Collier R.J., Preedy V.R. (2017). Chapter 16—Fermented Dairy Foods and Cardiovascular Risk a2—Watson, Ronald Ross. Dairy in Human Health and Disease Across the Lifespan.

[227] Rosqvist F., Smedman A., Lindmark-Månsson H., Paulsson M., Petrus P., Straniero S., Rudling M., Dahlman I., Risérus U. (2015). Potential role of milk fat globule membrane in modulating plasma lipoproteins, gene expression, and cholesterol metabolism in humans: A randomized study. Am. J. Clin. Nutr..

[228] Granato D., Branco G.F., Cruz A.G., Faria J.D.A.F., Shah N.P. (2010). Probiotic dairy products as functional foods. Compr. Rev. Food Sci. Food Saf..

[229] Shortt C., O’Brien J. (2016). Handbook of Functional Dairy Products.

[230] Vásquez-Trespalacios E.M., Romero-Palacio J. (2014). Efficacy of yogurt drink with added plant stanol esters (Benecol^®^, Colanta) in reducing total and LDL cholesterol in subjects with moderate hypercholesterolemia: A randomized placebo-controlled crossover trial NCT01461798. Lipids Health Dis..

[231] Law M. (2000). Plant sterol and stanol margarines and health. BMJ.

[232] Harland J.I. (2012). Food combinations for cholesterol lowering. Nutr. Res. Rev..

[233] Özer B.H., Kirmaci H.A. (2010). Functional milks and dairy beverages. Int. J. Dairy Technol..

[234] Rocha M., Banuls C., Bellod L., Jover A., M Victor V., Hernandez-Mijares A. (2011). A review on the role of phytosterols: New insights into cardiovascular risk. Curr. Pharm. Des..

[235] Yildiz F. (2016). Development and Manufacture of Yogurt and Other Functional Dairy Products.

[236] Jones P.J., Ntanios F.Y., Raeini-Sarjaz M., Vanstone C.A. (1999). Cholesterol-lowering efficacy of a sitostanol-containing phytosterol mixture with a prudent diet in hyperlipidemic men. Am. J. Clin. Nutr..

[237] Hansel B., Nicolle C., Lalanne F., Tondu F., Lassel T., Donazzolo Y., Ferrières J., Krempf M., Schlienger J.-L., Verges B. (2007). Effect of low-fat, fermented milk enriched with plant sterols on serum lipid profile and oxidative stress in moderate hypercholesterolemia. Am. J. Clin. Nutr..

[238] Mäkinen O.E., Wanhalinna V., Zannini E., Arendt E.K. (2016). Foods for special dietary needs: Non-dairy plant-based milk substitutes and fermented dairy-type products. Crit. Rev. Food Sci. Nutr..

[239] Stall S., Adams G. (2017). Can almond milk be called milk?. J. Renal Nutr..

[240] Jeske S., Zannini E., Arendt E.K. (2017). Evaluation of physicochemical and glycaemic properties of commercial plant-based milk substitutes. Plant Foods Hum. Nutr..

[241] Jenkins D.J., Mirrahimi A., Srichaikul K., Berryman C.E., Wang L., Carleton A., Abdulnour S., Sievenpiper J.L., Kendall C.W., Kris-Etherton P.M. (2010). Soy protein reduces serum cholesterol by both intrinsic and food displacement mechanisms. J. Nutr..

[242] Liu X.X., Li S.H., Chen J.Z., Sun K., Wang X.J., Wang X.G., Hui R.T. (2012). Effect of soy isoflavones on blood pressure: A meta-analysis of randomized controlled trials. Nutr. Metab. Cardiovasc. Dis..

[243] Messina M. (2016). Soy and health update: Evaluation of the clinical and epidemiologic literature. Nutrients.

[244] Lecomte M., Couëdelo L., Meugnier E., Plaisancié P., Létisse M., Benoit B., Gabert L., Penhoat A., Durand A., Pineau G. (2016). Dietary emulsifiers from milk and soybean differently impact adiposity and inflammation in association with modulation of colonic goblet cells in high-fat fed mice. Mol. Nutr. Food Res..

[245] O’Connor A. Got Almond Milk? Dairy Farms Protest Milk Label on Nondairy Drinks. https://www.nytimes.com/2017/02/13/well/eat/got-almond-milk-dairy-farms-protest-milk-label-on-nondairy-drinks.html?_r=0.

[246] Balthazar C., Junior C.C., Moraes J., Costa M., Raices R., Franco R., Cruz A., Silva A. (2016). Physicochemical evaluation of sheep milk yogurts containing different levels of inulin. J. Dairy Sci..

[247] Park Y.W. (2007). Impact of goat milk and milk products on human nutrition. Perspect. Agric. Vet. Sci. Nutr. Nat. Resour..

[248] Costa M.P., Monteiro M.L.G., Frasao B.S., Silva V.L.M., Rodrigues B.L., Chiappini C.C.J., Conte-Junior C.A. (2017). Consumer perception, health information, and instrumental parameters of cupuassu (*Theobroma grandiflorum*) goat milk yogurts. J. Dairy Sci..

[249] Park Y.W., Juárez M., Ramos M., Haenlein G.F.W. (2007). Physico-chemical characteristics of goat and sheep milk. Small Rumin. Res..

[250] Haenlein G.F.W. (2004). Goat milk in human nutrition. Small Rumin. Res..

[251] Pereira P.C. (2014). Milk nutritional composition and its role in human health. Nutrition.

[252] Contarini G., Pelizzola V., Povolo M. (2009). Content of conjugated linoleic acid in neutral and polar lipid fractions of milk of different ruminant species. Int. Dairy J..

[253] Pintus S., Murru E., Carta G., Cordeddu L., Batetta B., Accossu S., Pistis D., Uda S., Elena Ghiani M., Mele M. (2013). Sheep cheese naturally enriched in α-linolenic, conjugated linoleic and vaccenic acids improves the lipid profile and reduces anandamide in the plasma of hypercholesterolaemic subjects. Br. J. Nutr..

[254] Barłowska J., Szwajkowska M., Litwińczuk Z., Król J. (2011). Nutritional value and technological suitability of milk from various animal species used for dairy production. Compr. Rev. Food Sci. Food Saf..

[255] Haenlein G.F.W., Wendorff W.L. (2008). Sheep milk. Handbook of Milk of Non-Bovine Mammals.

[256] Balthazar C.F., Pimentel T.C., Ferrão L.L., Almada C.N., Santillo A., Albenzio M., Mollakhalili N., Mortazavian A.M., Nascimento J.S., Silva M.C. (2017). Sheep milk: Physicochemical characteristics and relevance for functional food development. Compr. Rev. Food Sci. Food Saf..

[257] Molkentin J. (2000). Occurrence and biochemical characteristics of natural bioactive substances in bovine milk lipids. Br. J. Nutr..

[258] Balthazar C.F., Cruz A.G. (2017). Sheep milk: An unexplored food matrix to develop functional foods. Inform.

[259] Corrêa A.P.F., Daroit D.J., Coelho J., Meira S.M.M., Lopes F.C., Segalin J., Risso P.H., Brandelli A. (2011). Antioxidant, antihypertensive and antimicrobial properties of ovine milk caseinate hydrolyzed with a microbial protease. J. Sci. Food Agric..

[260] Marsh A.J., O’Sullivan O., Hill C., Ross R.P., Cotter P.D. (2013). Sequencing-based analysis of the bacterial and fungal composition of *kefir* grains and milks from multiple sources. PLoS ONE.

[261] Farnworth E.R. (2006). *Kefir*—A complex probiotic. Food Sci. Technol. Bull..

[262] Vieira C., Álvares T., Gomes L., Torres A., Paschoalin V., Conte-Junior C. (2015). Kefir grains change fatty acid profile of milk during fermentation and storage. PLoS ONE.

[263] Bourrie B.C., Willing B.P., Cotter P.D. (2016). The microbiota and health promoting characteristics of the fermented beverage *kefir*. Front. Microbiol..

[264] Maeda H., Zhu X., Suzuki S., Suzuki K., Kitamura S. (2004). Structural characterization and biological activities of an exopolysaccharide kefiran produced by *Lactobacillus kefiranofaciens* Wt-2B^T^. J. Agric. Food Chem..

[265] Urdaneta E., Barrenetxe J., Aranguren P., Irigoyen A., Marzo F., Ibáñez F.C. (2007). Intestinal beneficial effects of *kefir*-supplemented diet in rats. Nutr. Res..

[266] St-Onge M.-P., Farnworth E.R., Savard T., Chabot D., Mafu A., Jones P.J. (2002). *Kefir* consumption does not alter plasma lipid levels or cholesterol fractional synthesis rates relative to milk in hyperlipidemic men: A randomized controlled trial (ISRCTN10820810). BMC Complement. Altern. Med..

[267] Ostadrahimi A., Taghizadeh A., Mobasseri M., Farrin N., Payahoo L., Beyramalipoor Gheshlaghi Z., Vahedjabbari M. (2015). Effect of probiotic fermented milk (*kefir*) on glycemic control and lipid profile in type 2 diabetic patients: A randomized double-blind placebo-controlled clinical trial. Iran. J. Public Health.

[268] Givens D.I. (2015). Dairy products: Good or bad for cardiometabolic disease?. Am. J. Clin. Nutr..

[269] Astrup A. (2014). Yogurt and dairy product consumption to prevent cardiometabolic diseases: Epidemiologic and experimental studies. Am. J. Clin. Nutr..

